# Clinicopathological significance of *CHFR* promoter methylation in gastric cancer: a meta-analysis

**DOI:** 10.18632/oncotarget.23394

**Published:** 2017-12-16

**Authors:** Yong Ding, Hai-Feng Lian, Yaowu Du

**Affiliations:** ^1^ School of Basic Medical Science, Henan University, Kaifeng, 475004, China; ^2^ Department of Gastroenterology, Affiliated Hospital of Binzhou Medical College, Binzhou, 256600, China; ^3^ Laboratory for Nanomedicine, School of Basic Medical Science, Henan University, Kaifeng, 475004, China

**Keywords:** CHFR, methylation, gastric cancer, biomarker, drug target

## Abstract

The mitotic checkpoint gene (*CHFR*) (Checkpoint with Forkhead-associated and Ring finger domains is a G2 phase/mitosis checkpoint and tumor-suppressor gene. Recent studies have reported the relationship of *CHFR* promoter methylation with clinicopathological significance of gastric cancer. However, the results remain unclear due to small size of sample. We pooled 15 studies including 827 gastric cancer patients and conducted a meta-analysis to investigate the clinicopathological significance of *CHFR* promoter methylation in gastric cancer. Our data revealed that the frequency of *CHFR* promoter methylation was higher in gastric cancer than in normal gastric tissue, Odd Ratio (OR) was 10.12 with 95% CI 5.17–19.79, *p* < 0.00001. Additionally, the rate of *CHFR* promoter methylation was significantly increased in high grade of gastric cancer compared to low grade, OR was 1.64 with 95% CI 1.00–2.68, *p* = 0.05. *CHFR* methylation was significantly associated with the positive lymph node metastasis, OR was 1.56 with 95% CI 1.05–2.32, *p* = 0.03. We concluded that *CHFR* could serve as a biomarker for diagnosis of gastric cancer, and a drug target for development of gene therapy in gastric cancer. *CHFR* promoter methylation is associated with tumor poor differentiation and lymph node metastasis.

## INTRODUCTION

Although gastric cancer (GC) incidence has significantly declined worldwide over the past a few decades, GC remains the fifth leading malignancy and the third most common cause of cancer-related mortality globally [1, 2]. GC etiology is multifactorial, including Helicobacter pylori and Epstein-Barr virus infections environmental risk factors [3]. In addition, genetic and epigenetic alterations of oncogenes and suppressor genes contributed to the initiation and development of GC.

*CHFR* gene is located at chromosome 12q24.33 and contains a forkhead and a RING finger domain. It functions as a cell-cycle checkpoint molecule by delaying entry into the metaphase in response to microtubule stress. *CHFR* gene is silenced by promoter hypermethylation or mutated in several primary tumors such as 20% in NSCLC [4], 30% in esophageal cancer [5], and 40% in colorectal cancer (CRC) [6]. The growing evidence supports its role as a tumor-suppressor protein and biomarker for chemotherapeutic response to microtubule-targeting drugs such as taxanes [7]. However, the rate of *CHFR* hypermethylation in GC was inconsistent and the relationship between CHFR methylation and clinicopathologic variables was unclear due to the small power of individual study. Our main objective was to systematically search and analyze the available studies regarding the clinicopathologic significance of *CHFR* promoter hypermethylation in GC.

## RESULTS

### Identification of relevant studies and quality assessment

**Figure 1:**
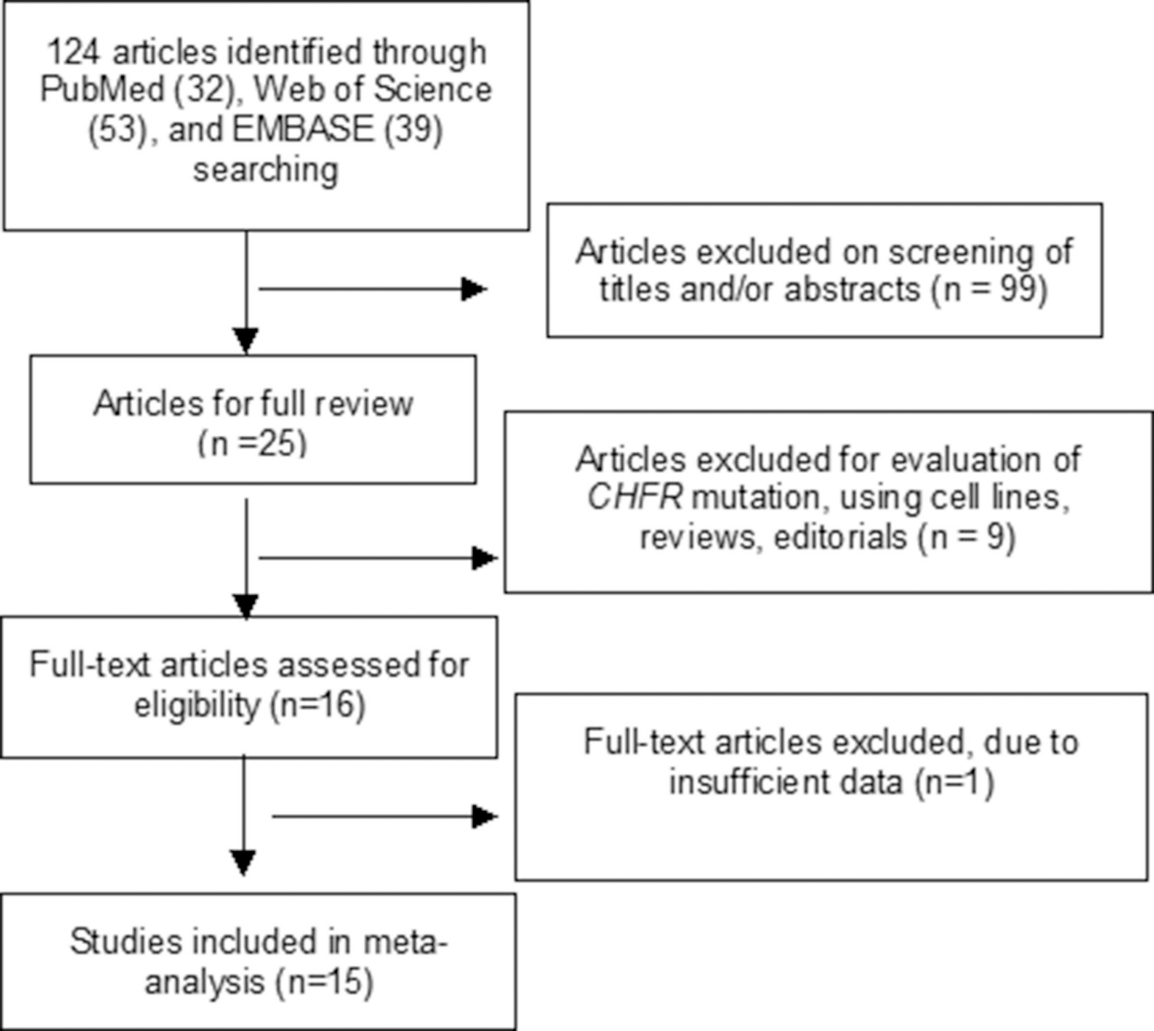
Schematic flow diagram for selection of included studies

A total of 15 studies satisfied the inclusion criteria and were included in current meta-analysis (Figure 1), 827 GC patients and 454 controls were enrolled. All studies included were retrospective observational cohort studies published from 2003 to 2015. The study characteristics were summarized in Table 1. Based on the quality evaluation with the NOQAS (Newcastle-Ottawa Quality Assessment Scale), the overall quality of 15 studies was scored from six to eight which indicated good quality (data not shown).

**Table 1:** Main characteristics of included studies

Author	Year	Country	Histology		Invasion		Grade		Stage(TNM)		LN		Method
			NCT	GC	Subserosa (-)	Subserosa(+)	L	H	Early	Advanced	-	+	
Li [30]	2015	China	-	35/102	7/13	28/89	9/29	26/73	6/19	29/83	10/36	25/66	MSP
Wang [31]	2014	China	0/46	35/117	-	-	-	-	-	-	-	-	MethyLight
Hiraki [32]	2011	Japan	1/20	13/20	-	-	-	-	-	-			MSP
Hu [33]	2010	China	16/70	34/70	-	-	8/25	26/45	11/31	23/39	7/23	27/47	MSP
Hiraki [34]	2010	Japan	15/49	31/49	20/33	11/18	-	-	16/24	15/25	11/15	20/34	MSP
Oki [35]	2009	Japan	6/59	20/59	8/23	12/36	-	-	5/17	15/42	5/20	15/39	MSP
Kang [36]	2008	Korea	0/25	11/25	-	-	-	-	-	-			MethyLight
Gao [37]	2008	China	0/20	9/20	2/5	7/15	0/6	9/14	4/10	5/10	3/8	6/12	MSP
Yoshida [38]	2006	Japan	0/41	15/41	-	-	-	-	-	-	-	-	COBRA
Mitsuno [39]	2007	Japan	-	23/56	-	-	6/12	10/26	0/4	16/34	4/10	12/28	MSP
Koga [15]	2006	Japan	2/46	24/46	-	-	-	-	-	-	-	-	MSP
Morioka [40]	2006	Japan	0/38	9/38	-	-	-	-	4/16	12/37	7/25	9/28	MSP
Homma [41]	2005	Japan	4/52	18/52	11/32	7/20	-	-	1/10	17/42	-	-	MSP
Honda [16]	2004	Japan	4/34	25/71	-	-	13/40	12/31	6/15	19/56	5/13	20/58	MSP
Satoh [17]	2003	Japan	0/44	24/61	-	-	-	-	-	-	-	-	COBRA

NCT: normal control tissue; GC: Gastric Cancer; L: low grade; H: high grade; LN: Lymph node, MSP: methylation-specific PCR, COBRA: Combined Bisulfite Restriction Analysis

### The frequency of CHFR promoter methylation in GC and normal gastric mucosa, and the association of CHFR methylation with the grade of GC, as well as the depth of invasion

**Figure 2:**
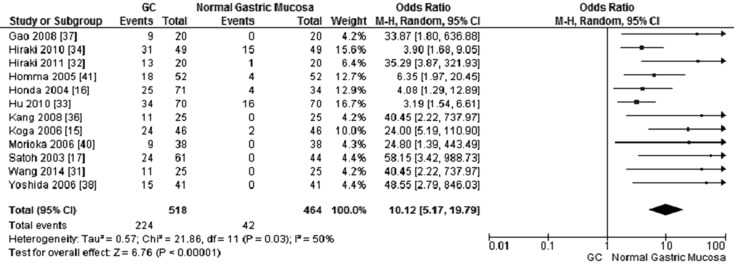
Forest plot for *CHFR* promoter methylation in GC and normal gastric tissue

**Figure 3:**
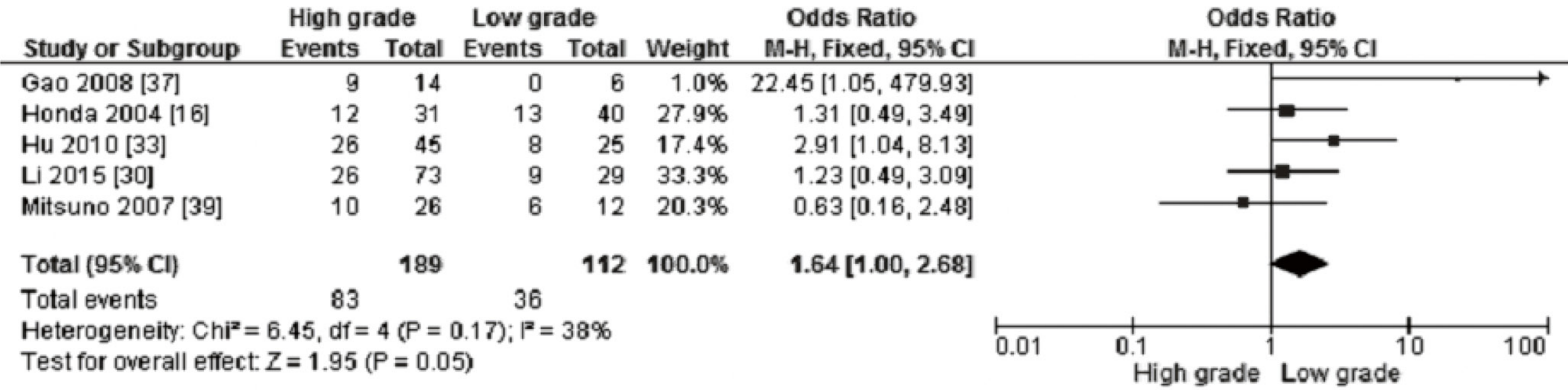
Forest plot for *CHFR* promoter methylation in different grade of GC

**Figure 4:**
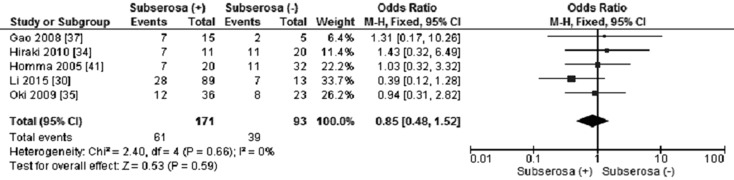
Forest plot for *CHFR* promoter methylation in different invasion status of GC

12 out of 15 studies reported the frequency of *CHFR* methylation in GC and normal gastric mucosa, as demonstrated in Figure 2, the frequency of *CHFR* methylation was significantly elevated in GC compared with normal gastric mucosa, the pooled OR was 10.12, with 95% CI 5.17–19.79, *p* < 0.00001, *I*^2^ = 50% (Figure 2). The rate of *CHFR* methylation in different grade of GC was compared, OR was 1.64, with 95% CI 1.00–2.68, *p* = 0.05, *I*^2^ = 38%, suggesting *CHFR* gene was more frequently methylated in high grade GC than in low grade GC (Figure 3). However, there was no association between the depth of invasion in GC patients and CHFR methylation, OR was 0.85, with 95% CI 0.48–1.52, *p* = 0.59, *I*^2^ = 0% (Figure 4).

### The relationship of CHFR promoter methylation with stage of GC and status of lymph node metastasis

**Figure 5:**
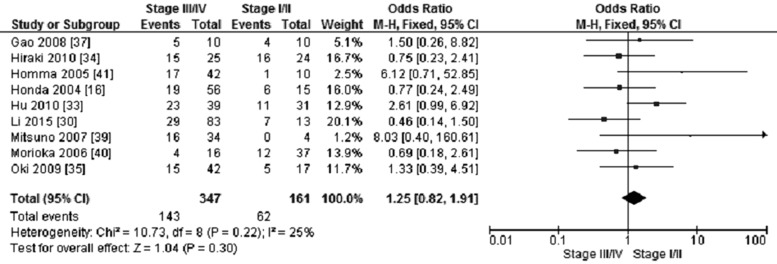
Forest plot for *CHFR* promoter methylation in GC stage III/IV and stage I/II

**Figure 6:**
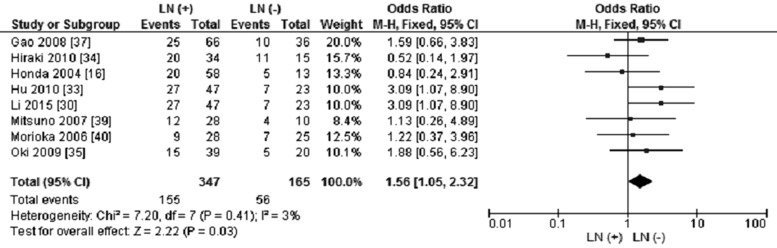
Forest plot for *CHFR* promoter methylation in different status of lymph node metastasis

*CHFR* promoter methylation was not correlated with GC stages, the frequency of *CHFR* promoter methylation in stage III/IV GC was not significantly increased compared to stage I/II GC, OR was 1.25, 95% CI 0.82–1.91, *p* = 0.30 (Figure 5). The frequency of *CHFR* methylation in different status of lymph node metastasis was evaluated, our findings demonstrated that the *CHFR* methylation occurred more frequently in GC patients with lymph node metastasis in contrast to the patient without lymph node metastasis. The pooled OR was 1.56, with 95% CI 1.05–2.32, *p* = 0.03, *I*^2^ = 3% (Figure 6).

### Sensitivity analysis and publication bias

**Figure 7:**
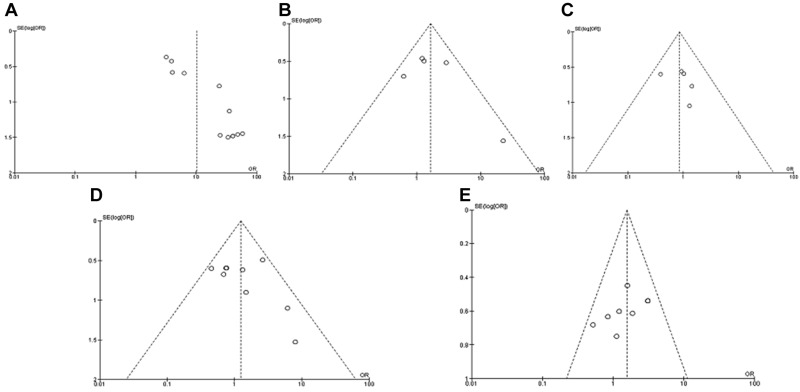
Funnel plot for publication bias (**A**) *CHFR* promoter methylation in GC and normal gastric tissue; (**B**) *CHFR* promoter methylation in high and low grade of GC; (**C**) *CHFR* promoter methylation in different invasion status of GC; (**D**) *CHFR* promoter methylation in GC stage III/IV and stage I/II; (**E**) *CHFR* promoter methylation in different status of lymph node metastasis.

A sensitivity analysis was conducted by omitting one study at a time, the ORs were not significantly changed, indicating the stability of present meta-analysis (Supplementary Figures 1–5). The shape of the funnel plots were largely symmetric (Figure 7), suggesting there was no publication biases existed in the meta-analysis of association between *CHFR* promoter methylation and clinicopathological variables.

## DISCUSSION

The lack of *CHFR* gene was initially observed in colorectal cancer and neuroblastoma cell lines by Scolnik and Halazonetis, then loss of CHFR expression has been reported in a number of malignancies including colorectal cancer [6, 8, 9], esophageal cancer [5, 10], NSCLC[11–13] and gastric cancer [14–17]. Previous evidence indicated *CHFR* was mostly inactivated by its promotor CpG island methylation [18]. *CHFR* promoter methylation has been evaluated in GC, however, the frequency was inconsistent due to small size of samples. We pooled 12 studies together and compared the frequency of *CHFR* promoter methylation in 827 GC with 454 non-malignancy gastric mucosa, the result indicated that *CHFR* promoter methylation in GC was 10.12 times higher than in non-malignancy gastric mucosa. Our result was consistent with previous meta-analysis [19]. Previous evidence indicated that CHFR ubiquitinates and binds to both polo-like-kinase (PLK1) and Aurora A, results in the inhibition of phosphorylation of Cdc25 [20–23]. The cyclin B1-Cdk complex is not able to form and the cell cycle is arrested in G2 phase [23, 24]. Thus, cells with *CHFR* gene inactivated by promoter methylation cannot be arrested in the G2 phase and proceed to mitosis, leading to abnormal proliferation and differentiation. Therefore, CHFR promoter methylation is associated with the risk of GC incidence.

Further subgroup analysis revealed that *CHFR* promoter methylation was associated with poor differentiation of GC. However, the mechanism is unclear and further investigation needs to be finished in future.

Additionally, our finding indicated that *CHFR* promoter methylation was strongly associated with lymph node metastasis. Previous evidence showed that abnormal CHFR expression down-regulated histone deacetylase 1 and promoted the expression of *p21*^CIP1/WAF1^ and metastasis suppressors kangai 1 and E-cadherin [25]. Recent studies indicated that reduced kangai 1 expression was associated with lymph node metastasis [26]. Additionally, CHFR act as a negative regulator of the nuclear factor kB pathway, whereas kB activation contributes to lymph node metastasis [27, 28]. Thus, *CHFR* promoter methylation is correlated with lymph node metastasis in GC.

A few limitations of this meta-analysis should be noted. First, present findings were based on the patients from Asia, cautions should be taken when the findings are interpreted among the general populations. Second, publication bias may exist, as positive results were more likely published. Third, the possibility of information and selection biases as well as unidentified confounders could not be completely excluded because all of the included studies were observational.

In summary, *CHFR* promoter methylation is associated with the risk of GC development. *CHFR* could be a potential biomarker for diagnosis and a drug target of personalized treatment for the patients with GC. *CHFR* promoter methylation is correlated with GC poor differentiation and positive lymph node metastasis.

## MATERIALS AND METHODS

### Study identification

We performed a systematic literature search for articles published from the earliest available data to November 2017 in PubMed, EMBASE, and Web of Science with no limit set for date and language. The search terms were “*CHFR*, or Checkpoint with Forkhead-associated and Ring finger domains” and “gastric cancer”, “GC”, “methylation”. We conducted a manual search and reviewed the reference lists of include studies for any further relevant citations.

After screening by titles and abstracts, individual studies were screened using the inclusion and exclusion criteria. We included studies that met the following criteria: 1) *CHFR* hypermethylation evaluated in GC tissue; 2) Study revealed the relationship between *CHFR* hypermethylation and GC clinicopathological variables. Exclusion criteria included the following: 1) studies using cell line and human xenografts, as well as using the same population and overlapping database. 2) reviews, conference abstracts, editorials, letters, case reports, and expert opinion. The flow chart of searches was shown in Figure 1.

### Data extraction

Primary data were extracted by using a customized form that included first author, year of publication, geography location, the number of case, the depth of invasion, GC TNM stages, grades and methylation detect methods. Two authors independently extracted data, any discrepancies were discussed until a consensus was reached as a first step, then by consultation with the senior study investigator if consensus was not reached.

### Quality assessment

The methodological quality of included studies was evaluated based on NOQAS. This scale was used to allocate a maximum of nine points, 0–4 points for selection, 0–2 points for comparability, 0–3 points for outcomes. The NOS scores ranged from 0 to 9, and a score ≥ 7 indicates a good quality. All studies were assessed by HL and YDu independently, any disagreements were discussed until a consensus was reached.

### Statistical analysis

The pooled ORs with its 95% confidence intervals were calculated. The heterogeneity among studies was determined by using the Cochran’s Q statistic and *I*^2^ tests. When the *I*^2^ value was below 50%, fixed effect model was used, when the *I*^2^ value was 50% or greater, a random effect model was used. An Egger’s test for asymmetry of funnel was used to assess for publication bias [29]. The analysis was performed to compare the frequency of *CHFR* methylation between GC and normal gastric mucosa. The frequency of *CHFR* hypermethylation was compared in different tumor characteristics. The pooled ORs were estimated for the correlation between *CHFR* hypermethylation and clinicopathological features. The meta-analysis was performed using Review Manager 5.3 (Cochrane Collaboration, Software Update, Oxford, UK). *P* values tailed less than 0.05 were considered statistically significant.

## SUPPLEMENTARY MATERIALS FIGURES


