# Supplementary material for: Clinicopathological significance of CHFR promoter methylation in gastric cancer: a meta-analysis
Source: Oncotarget. 2017 Dec 16;9(11):10083–90. doi: 10.18632/oncotarget.23394 (PMC5839373; doi:10.18632/oncotarget.23394)
Supplement: Supplementary file 1 [file oncotarget-09-10083-s001.pdf]

Clinicopathological significance of *CHFR* promoter methylation in gastric cancer: a meta-analysis

SUPPLEMENTARY MATERIALS

Study or Subgroup	GC Events	GC Total	Normal Gastric Mucosa Events	Normal Gastric Mucosa Total	Weight	Odds Ratio M-H, Random, 95% CI
Gao 2008 [1]	9	20	0	20	4.2%	33.87 [1.80, 636.88]
Hiraki 2010 [2]	31	49	15	49	15.6%	3.90 [1.68, 9.05]
Hiraki 2011 [3]	13	20	1	20	6.4%	35.29 [3.87, 321.93]
Homma 2005 [4]	18	52	4	52	12.7%	6.35 [1.97, 20.45]
Honda 2004 [5]	25	71	4	34	12.9%	4.08 [1.29, 12.89]
Hu 2010 [6]	34	70	16	70	16.7%	3.19 [1.54, 6.61]
Kang 2008 [7]	11	25	0	25	4.2%	40.45 [2.22, 737.97]
Koga 2006 [8]	24	46	2	46	10.0%	24.00 [5.19, 110.90]
Morioka 2006 [9]	9	38	0	38	4.3%	24.80 [1.39, 443.49]
Satoh 2003 [10]	24	61	0	44	4.4%	58.15 [3.42, 988.73]
Wang 2014 [11]	11	25	0	25	4.2%	40.45 [2.22, 737.97]
Yoshida 2006 [12]	15	41	0	41	4.4%	48.55 [2.79, 846.03]
**Total (95% CI)**		**518**		**464**	**100.0%**	**10.12 [5.17, 19.79]**
Total events	224		42			

Heterogeneity: Tau² = 0.57; Chi² = 21.86, df = 11 (P = 0.03); I² = 50%
Test for overall effect: Z = 6.76 (P < 0.00001)

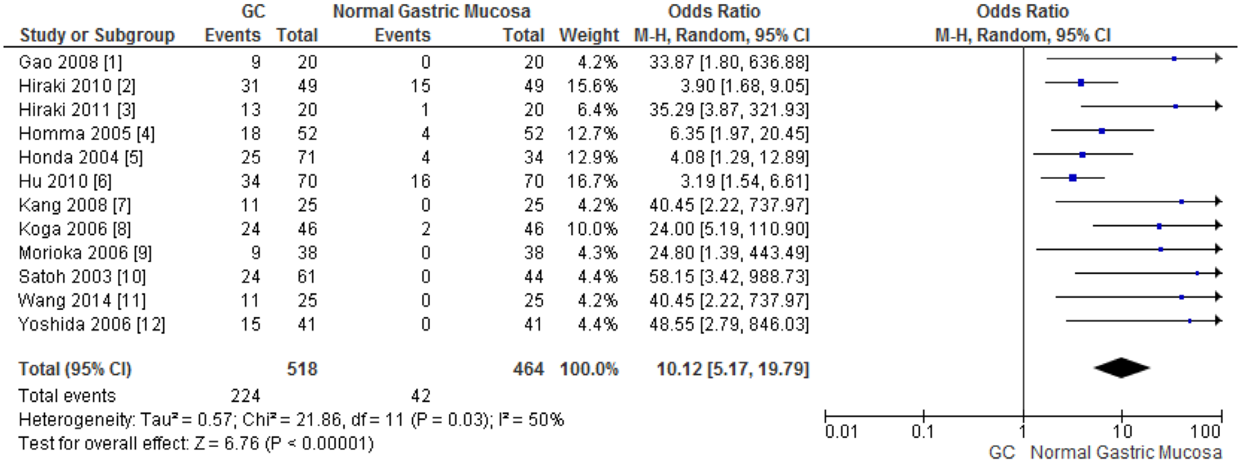

Study or Subgroup	GC Events	GC Total	Normal Gastric Mucosa Events	Normal Gastric Mucosa Total	Weight	Odds Ratio M-H, Random, 95% CI
Hiraki 2010 [2]	31	49	15	49	16.4%	3.90 [1.68, 9.05]
Hiraki 2011 [3]	13	20	1	20	6.6%	35.29 [3.87, 321.93]
Homma 2005 [4]	18	52	4	52	13.3%	6.35 [1.97, 20.45]
Honda 2004 [5]	25	71	4	34	13.4%	4.08 [1.29, 12.89]
Hu 2010 [6]	34	70	16	70	17.4%	3.19 [1.54, 6.61]
Kang 2008 [7]	11	25	0	25	4.4%	40.45 [2.22, 737.97]
Koga 2006 [8]	24	46	2	46	10.4%	24.00 [5.19, 110.90]
Morioka 2006 [9]	9	38	0	38	4.5%	24.80 [1.39, 443.49]
Satoh 2003 [10]	24	61	0	44	4.6%	58.15 [3.42, 988.73]
Wang 2014 [11]	11	25	0	25	4.4%	40.45 [2.22, 737.97]
Yoshida 2006 [12]	15	41	0	41	4.5%	48.55 [2.79, 846.03]
**Total (95% CI)**		**498**		**444**	**100.0%**	**9.58 [4.84, 18.97]**
Total events	215		42			

Heterogeneity: Tau² = 0.56; Chi² = 20.46, df = 10 (P = 0.03); I² = 51%
Test for overall effect: Z = 6.48 (P < 0.00001)

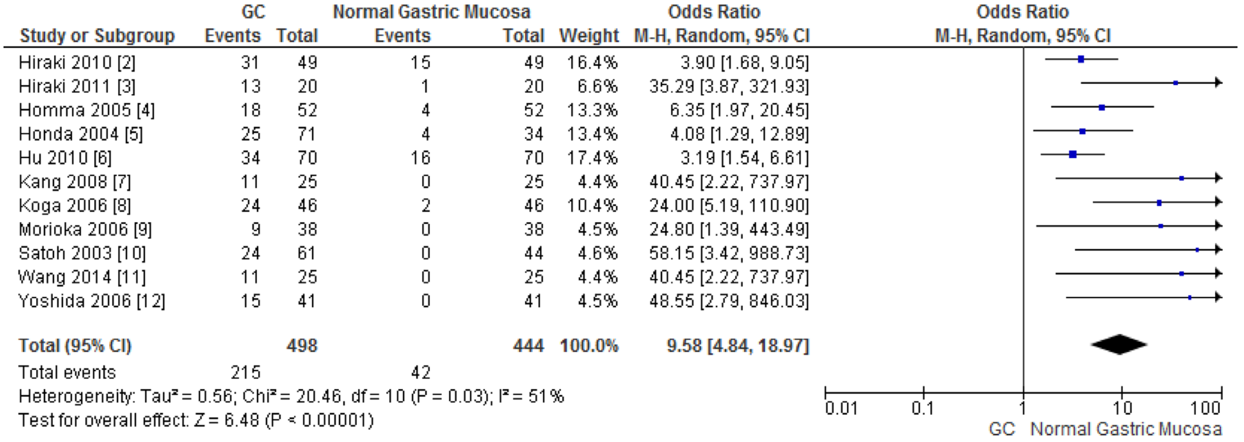

Study or Subgroup	GC Events	GC Total	Normal Gastric Mucosa Events	Normal Gastric Mucosa Total	Weight	Odds Ratio M-H, Random, 95% CI
Gao 2008 [1]	9	20	0	20	5.3%	33.87 [1.80, 636.88]
Hiraki 2011 [3]	13	20	1	20	7.9%	35.29 [3.87, 321.93]
Homma 2005 [4]	18	52	4	52	14.6%	6.35 [1.97, 20.45]
Honda 2004 [5]	25	71	4	34	14.8%	4.08 [1.29, 12.89]
Hu 2010 [6]	34	70	16	70	18.4%	3.19 [1.54, 6.61]
Kang 2008 [7]	11	25	0	25	5.4%	40.45 [2.22, 737.97]
Koga 2006 [8]	24	46	2	46	11.8%	24.00 [5.19, 110.90]
Morioka 2006 [9]	9	38	0	38	5.4%	24.80 [1.39, 443.49]
Satoh 2003 [10]	24	61	0	44	5.6%	58.15 [3.42, 988.73]
Wang 2014 [11]	11	25	0	25	5.4%	40.45 [2.22, 737.97]
Yoshida 2006 [12]	15	41	0	41	5.5%	48.55 [2.79, 846.03]
**Total (95% CI)**		**469**		**415**	**100.0%**	**12.73 [5.87, 27.58]**
Total events	193		27			

Heterogeneity: Tau² = 0.71; Chi² = 19.97, df = 10 (P = 0.03); I² = 50%
Test for overall effect: Z = 6.45 (P < 0.00001)

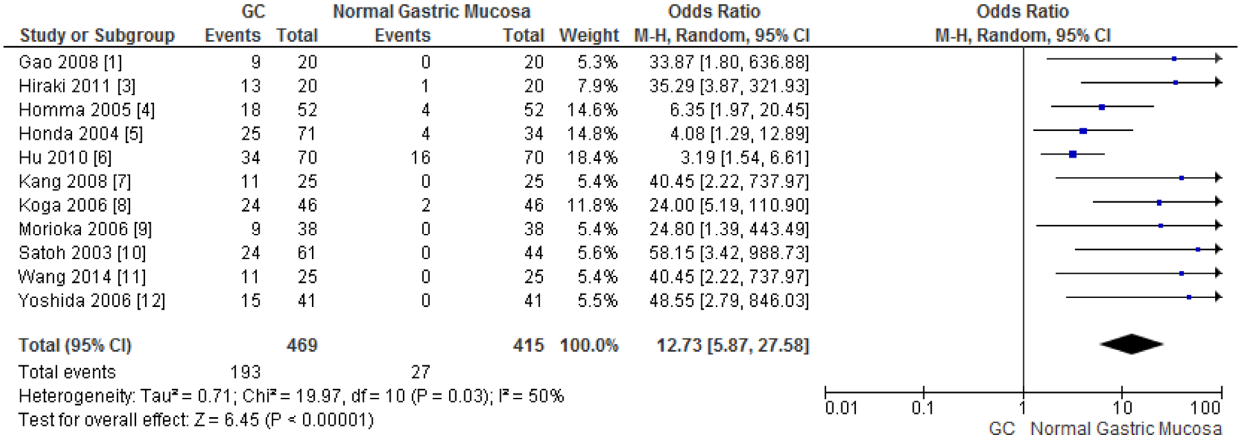

	GC		Normal Gastric Mucosa			Odds Ratio
Study or Subgroup	Events	Total	Events	Total	Weight	M-H, Random, 95% CI
Gao 2008 [1]	9	20	0	20	5.2%	33.87 [1.80, 636.88]
Hiraki 2010 [2]	31	49	15	49	16.8%	3.90 [1.68, 9.05]
Hiraki 2011 [3]	13	20	1	20	7.7%	35.29 [3.87, 321.93]
Honda 2004 [5]	25	71	4	34	14.3%	4.08 [1.29, 12.89]
Hu 2010 [6]	34	70	16	70	17.7%	3.19 [1.54, 6.61]
Kang 2008 [7]	11	25	0	25	5.3%	40.45 [2.22, 737.97]
Koga 2006 [8]	24	46	2	46	11.5%	24.00 [5.19, 110.90]
Morioka 2006 [9]	9	38	0	38	5.3%	24.80 [1.39, 443.49]
Satoh 2003 [10]	24	61	0	44	5.5%	58.15 [3.42, 988.73]
Wang 2014 [11]	11	25	0	25	5.3%	40.45 [2.22, 737.97]
Yoshida 2006 [12]	15	41	0	41	5.4%	48.55 [2.79, 846.03]
**Total (95% CI)**		**466**		**412**	**100.0%**	**11.64 [5.38, 25.22]**
Total events	206		38			

Heterogeneity: Tau² = 0.74; Chi² = 22.02, df = 10 (P = 0.02); I² = 55%
Test for overall effect: Z = 6.23 (P < 0.00001)

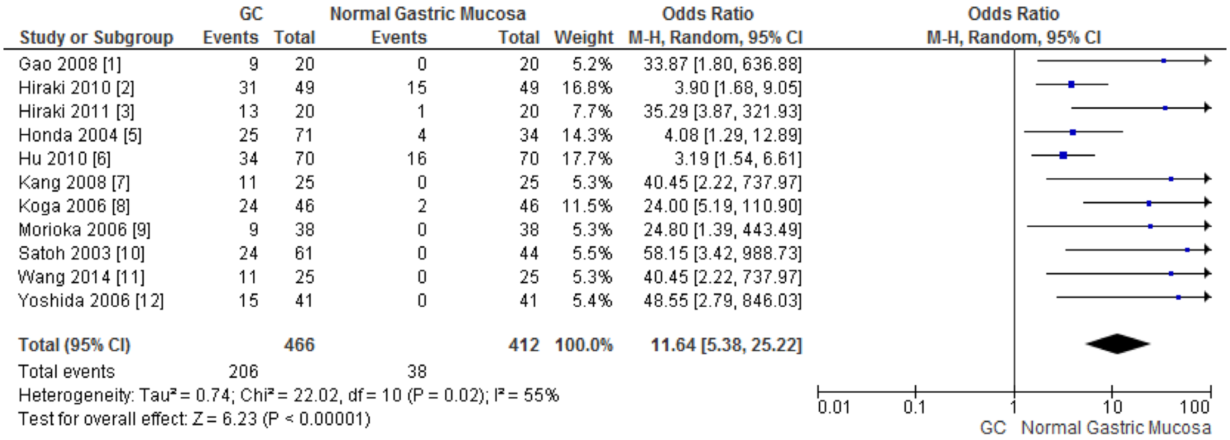

	GC		Normal Gastric Mucosa			Odds Ratio
Study or Subgroup	Events	Total	Events	Total	Weight	M-H, Random, 95% CI
Gao 2008 [1]	9	20	0	20	5.1%	33.87 [1.80, 636.88]
Hiraki 2010 [2]	31	49	15	49	17.0%	3.90 [1.68, 9.05]
Hiraki 2011 [3]	13	20	1	20	7.7%	35.29 [3.87, 321.93]
Homma 2005 [4]	18	52	4	52	14.3%	6.35 [1.97, 20.45]
Hu 2010 [6]	34	70	16	70	18.0%	3.19 [1.54, 6.61]
Kang 2008 [7]	11	25	0	25	5.2%	40.45 [2.22, 737.97]
Koga 2006 [8]	24	46	2	46	11.5%	24.00 [5.19, 110.90]
Morioka 2006 [9]	9	38	0	38	5.3%	24.80 [1.39, 443.49]
Satoh 2003 [10]	24	61	0	44	5.4%	58.15 [3.42, 988.73]
Wang 2014 [11]	11	25	0	25	5.2%	40.45 [2.22, 737.97]
Yoshida 2006 [12]	15	41	0	41	5.3%	48.55 [2.79, 846.03]
**Total (95% CI)**		**447**		**430**	**100.0%**	**12.25 [5.72, 26.25]**
Total events	199		38			

Heterogeneity: Tau² = 0.70; Chi² = 21.34, df = 10 (P = 0.02); I² = 53%
Test for overall effect: Z = 6.44 (P < 0.00001)

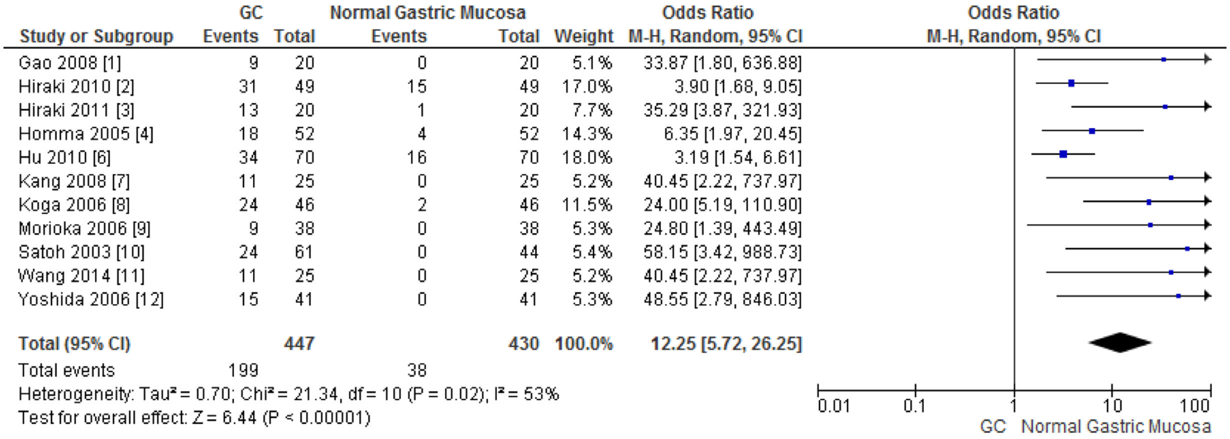

	GC		Normal Gastric Mucosa			Odds Ratio
Study or Subgroup	Events	Total	Events	Total	Weight	M-H, Random, 95% CI
Gao 2008 [1]	9	20	0	20	4.8%	33.87 [1.80, 636.88]
Hiraki 2010 [2]	31	49	15	49	19.5%	3.90 [1.68, 9.05]
Hiraki 2011 [3]	13	20	1	20	7.5%	35.29 [3.87, 321.93]
Homma 2005 [4]	18	52	4	52	15.6%	6.35 [1.97, 20.45]
Honda 2004 [5]	25	71	4	34	15.8%	4.08 [1.29, 12.89]
Kang 2008 [7]	11	25	0	25	4.9%	40.45 [2.22, 737.97]
Koga 2006 [8]	24	46	2	46	12.0%	24.00 [5.19, 110.90]
Morioka 2006 [9]	9	38	0	38	5.0%	24.80 [1.39, 443.49]
Satoh 2003 [10]	24	61	0	44	5.1%	58.15 [3.42, 988.73]
Wang 2014 [11]	11	25	0	25	4.9%	40.45 [2.22, 737.97]
Yoshida 2006 [12]	15	41	0	41	5.0%	48.55 [2.79, 846.03]
**Total (95% CI)**		**448**		**394**	**100.0%**	**12.37 [6.08, 25.17]**
Total events	190		26			

Heterogeneity: Tau² = 0.49; Chi² = 16.47, df = 10 (P = 0.09); I² = 39%
Test for overall effect: Z = 6.94 (P < 0.00001)

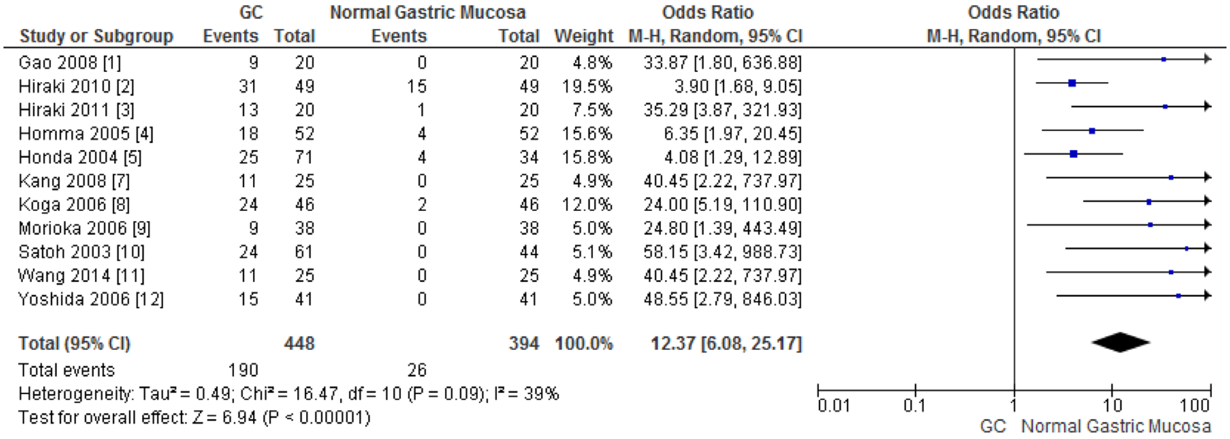

Study or Subgroup	GC Events	GC Total	Normal Gastric Mucosa Events	Normal Gastric Mucosa Total	Weight	Odds Ratio M-H, Random, 95% CI
Gao 2008 [1]	9	20	0	20	4.3%	33.87 [1.80, 636.88]
Hiraki 2010 [2]	31	49	15	49	16.5%	3.90 [1.68, 9.05]
Hiraki 2011 [3]	13	20	1	20	6.6%	35.29 [3.87, 321.93]
Homma 2005 [4]	18	52	4	52	13.3%	6.35 [1.97, 20.45]
Honda 2004 [5]	25	71	4	34	13.5%	4.08 [1.29, 12.89]
Hu 2010 [6]	34	70	16	70	17.6%	3.19 [1.54, 6.61]
Koga 2006 [8]	24	46	2	46	10.4%	24.00 [5.19, 110.90]
Morioka 2006 [9]	9	38	0	38	4.4%	24.80 [1.39, 443.49]
Satoh 2003 [10]	24	61	0	44	4.5%	58.15 [3.42, 988.73]
Wang 2014 [11]	11	25	0	25	4.4%	40.45 [2.22, 737.97]
Yoshida 2006 [12]	15	41	0	41	4.5%	48.55 [2.79, 846.03]
**Total (95% CI)**		**493**		**439**	**100.0%**	**9.41 [4.78, 18.52]**
Total events	213		42			

Heterogeneity: Tau² = 0.54; Chi² = 20.05, df = 10 (P = 0.03); I² = 50%
Test for overall effect: Z = 6.49 (P < 0.00001)

Odds Ratio
M-H, Random, 95% CI

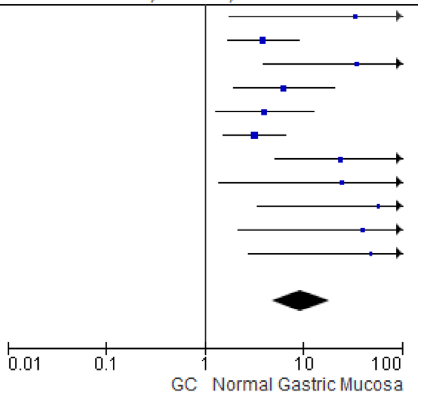

Study or Subgroup	GC Events	GC Total	Normal Gastric Mucosa Events	Normal Gastric Mucosa Total	Weight	Odds Ratio M-H, Random, 95% CI
Gao 2008 [1]	9	20	0	20	4.5%	33.87 [1.80, 636.88]
Hiraki 2010 [2]	31	49	15	49	17.8%	3.90 [1.68, 9.05]
Hiraki 2011 [3]	13	20	1	20	6.9%	35.29 [3.87, 321.93]
Homma 2005 [4]	18	52	4	52	14.3%	6.35 [1.97, 20.45]
Honda 2004 [5]	25	71	4	34	14.4%	4.08 [1.29, 12.89]
Hu 2010 [6]	34	70	16	70	19.1%	3.19 [1.54, 6.61]
Kang 2008 [7]	11	25	0	25	4.5%	40.45 [2.22, 737.97]
Morioka 2006 [9]	9	38	0	38	4.6%	24.80 [1.39, 443.49]
Satoh 2003 [10]	24	61	0	44	4.7%	58.15 [3.42, 988.73]
Wang 2014 [11]	11	25	0	25	4.5%	40.45 [2.22, 737.97]
Yoshida 2006 [12]	15	41	0	41	4.7%	48.55 [2.79, 846.03]
**Total (95% CI)**		**472**		**418**	**100.0%**	**8.93 [4.50, 17.72]**
Total events	200		40			

Heterogeneity: Tau² = 0.50; Chi² = 18.74, df = 10 (P = 0.04); I² = 47%
Test for overall effect: Z = 6.26 (P < 0.00001)

Odds Ratio
M-H, Random, 95% CI

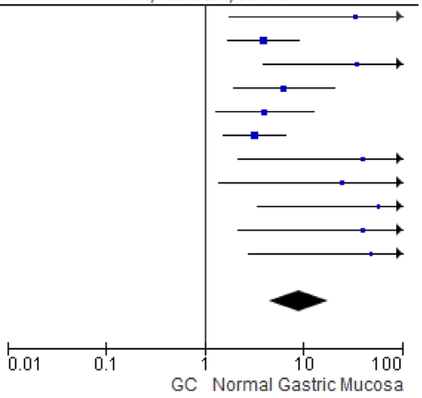

Study or Subgroup	GC Events	GC Total	Normal Gastric Mucosa Events	Normal Gastric Mucosa Total	Weight	Odds Ratio M-H, Random, 95% CI
Gao 2008 [1]	9	20	0	20	4.4%	33.87 [1.80, 636.88]
Hiraki 2010 [2]	31	49	15	49	16.3%	3.90 [1.68, 9.05]
Hiraki 2011 [3]	13	20	1	20	6.7%	35.29 [3.87, 321.93]
Homma 2005 [4]	18	52	4	52	13.3%	6.35 [1.97, 20.45]
Honda 2004 [5]	25	71	4	34	13.4%	4.08 [1.29, 12.89]
Hu 2010 [6]	34	70	16	70	17.3%	3.19 [1.54, 6.61]
Kang 2008 [7]	11	25	0	25	4.5%	40.45 [2.22, 737.97]
Koga 2006 [8]	24	46	2	46	10.4%	24.00 [5.19, 110.90]
Satoh 2003 [10]	24	61	0	44	4.7%	58.15 [3.42, 988.73]
Wang 2014 [11]	11	25	0	25	4.5%	40.45 [2.22, 737.97]
Yoshida 2006 [12]	15	41	0	41	4.6%	48.55 [2.79, 846.03]
**Total (95% CI)**		**480**		**426**	**100.0%**	**9.78 [4.90, 19.53]**
Total events	215		42			

Heterogeneity: Tau² = 0.58; Chi² = 20.85, df = 10 (P = 0.02); I² = 52%
Test for overall effect: Z = 6.47 (P < 0.00001)

Odds Ratio
M-H, Random, 95% CI

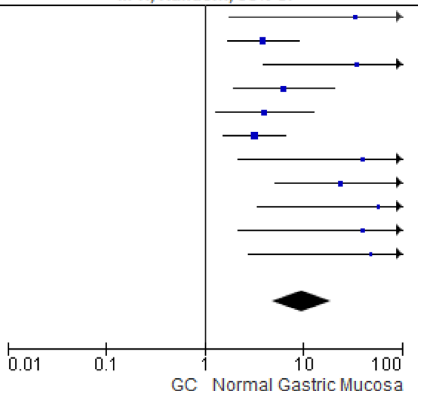

Study or Subgroup	GC Events	GC Total	Normal Gastric Mucosa Events	Normal Gastric Mucosa Total	Weight	Odds Ratio M-H, Random, 95% CI
Gao 2008 [1]	9	20	0	20	4.1%	33.87 [1.80, 636.88]
Hiraki 2010 [2]	31	49	15	49	17.0%	3.90 [1.68, 9.05]
Hiraki 2011 [3]	13	20	1	20	6.4%	35.29 [3.87, 321.93]
Homma 2005 [4]	18	52	4	52	13.5%	6.35 [1.97, 20.45]
Honda 2004 [5]	25	71	4	34	13.6%	4.08 [1.29, 12.89]
Hu 2010 [6]	34	70	16	70	18.3%	3.19 [1.54, 6.61]
Kang 2008 [7]	11	25	0	25	4.2%	40.45 [2.22, 737.97]
Koga 2006 [8]	24	46	2	46	10.3%	24.00 [5.19, 110.90]
Morioka 2006 [9]	9	38	0	38	4.2%	24.80 [1.39, 443.49]
Wang 2014 [11]	11	25	0	25	4.2%	40.45 [2.22, 737.97]
Yoshida 2006 [12]	15	41	0	41	4.3%	48.55 [2.79, 846.03]
**Total (95% CI)**		**457**		**420**	**100.0%**	**8.97 [4.66, 17.25]**
Total events	200		42			

Heterogeneity: Tau² = 0.47; Chi² = 18.79, df = 10 (P = 0.04); I² = 47%
Test for overall effect: Z = 6.58 (P < 0.00001)

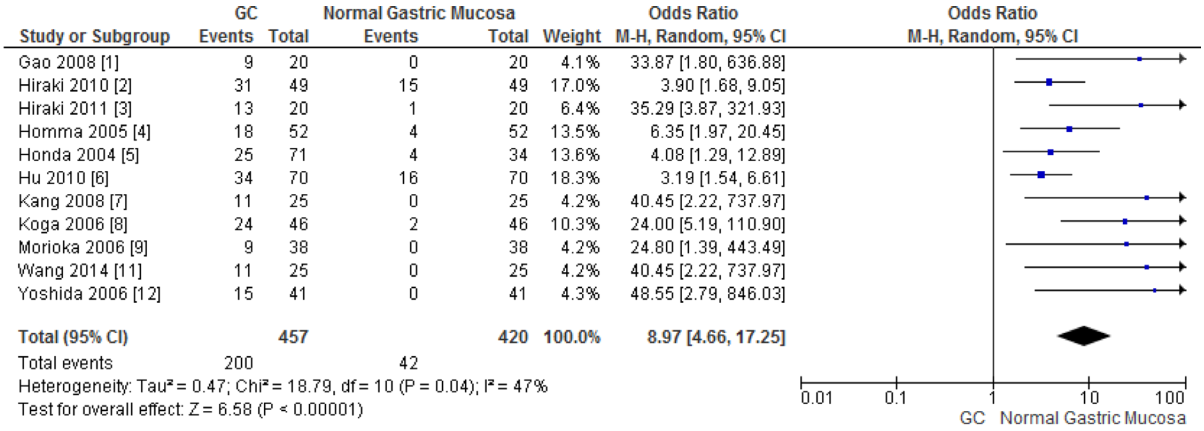

Study or Subgroup	GC Events	GC Total	Normal Gastric Mucosa Events	Normal Gastric Mucosa Total	Weight	Odds Ratio M-H, Random, 95% CI
Gao 2008 [1]	9	20	0	20	4.3%	33.87 [1.80, 636.88]
Hiraki 2010 [2]	31	49	15	49	16.5%	3.90 [1.68, 9.05]
Hiraki 2011 [3]	13	20	1	20	6.6%	35.29 [3.87, 321.93]
Homma 2005 [4]	18	52	4	52	13.3%	6.35 [1.97, 20.45]
Honda 2004 [5]	25	71	4	34	13.5%	4.08 [1.29, 12.89]
Hu 2010 [6]	34	70	16	70	17.6%	3.19 [1.54, 6.61]
Kang 2008 [7]	11	25	0	25	4.4%	40.45 [2.22, 737.97]
Koga 2006 [8]	24	46	2	46	10.4%	24.00 [5.19, 110.90]
Morioka 2006 [9]	9	38	0	38	4.4%	24.80 [1.39, 443.49]
Satoh 2003 [10]	24	61	0	44	4.5%	58.15 [3.42, 988.73]
Wang 2014 [11]	11	25	0	25		40.45 [2.22, 737.97]
Yoshida 2006 [12]	15	41	0	41	4.5%	48.55 [2.79, 846.03]
**Total (95% CI)**		**493**		**439**	**100.0%**	**9.41 [4.78, 18.52]**
Total events	213		42			

Heterogeneity: Tau² = 0.54; Chi² = 20.05, df = 10 (P = 0.03); I² = 50%
Test for overall effect: Z = 6.49 (P < 0.00001)

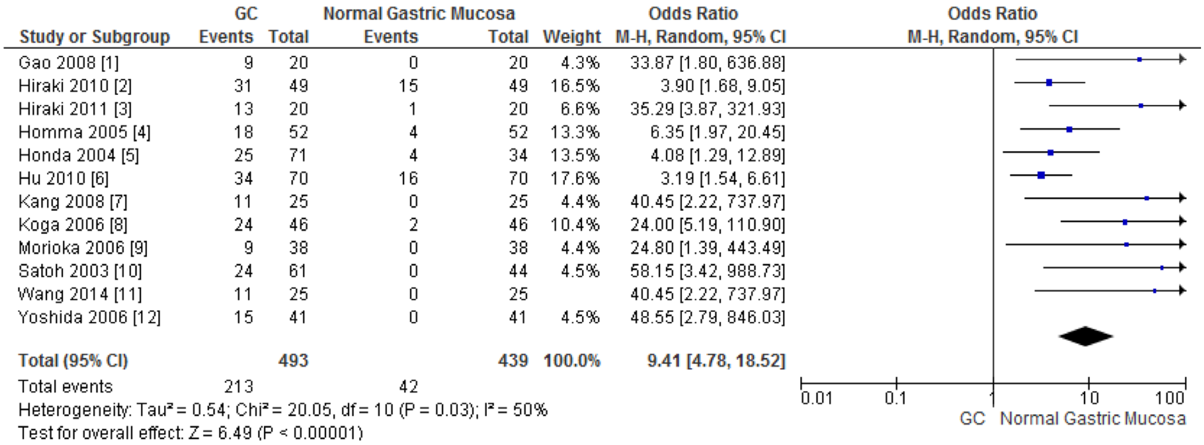

Study or Subgroup	GC Events	GC Total	Normal Gastric Mucosa Events	Normal Gastric Mucosa Total	Weight	Odds Ratio M-H, Random, 95% CI
Gao 2008 [1]	9	20	0	20	4.2%	33.87 [1.80, 636.88]
Hiraki 2010 [2]	31	49	15	49	16.7%	3.90 [1.68, 9.05]
Hiraki 2011 [3]	13	20	1	20	6.5%	35.29 [3.87, 321.93]
Homma 2005 [4]	18	52	4	52	13.4%	6.35 [1.97, 20.45]
Honda 2004 [5]	25	71	4	34	13.6%	4.08 [1.29, 12.89]
Hu 2010 [6]	34	70	16	70	17.9%	3.19 [1.54, 6.61]
Kang 2008 [7]	11	25	0	25	4.3%	40.45 [2.22, 737.97]
Koga 2006 [8]	24	46	2	46	10.4%	24.00 [5.19, 110.90]
Morioka 2006 [9]	9	38	0	38	4.3%	24.80 [1.39, 443.49]
Satoh 2003 [10]	24	61	0	44	4.5%	58.15 [3.42, 988.73]
Wang 2014 [11]	11	25	0	25	4.3%	40.45 [2.22, 737.97]
**Total (95% CI)**		**477**		**423**	**100.0%**	**9.20 [4.72, 17.91]**
Total events	209		42			

Heterogeneity: Tau² = 0.51; Chi² = 19.46, df = 10 (P = 0.03); I² = 49%
Test for overall effect: Z = 6.53 (P < 0.00001)

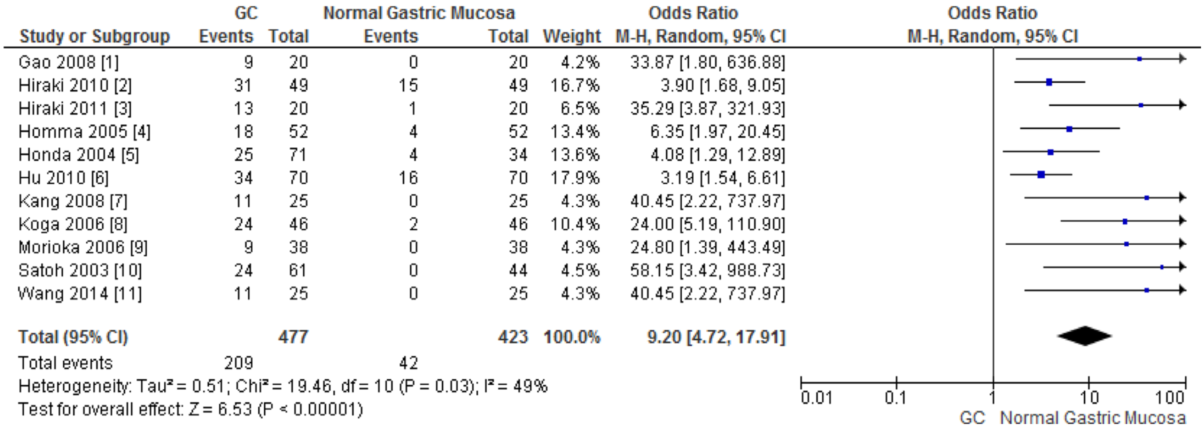

Supplementary Figure 1: Sensitivity analysis for *CHFR* promoter methylation in GC and normal gastric tissue.

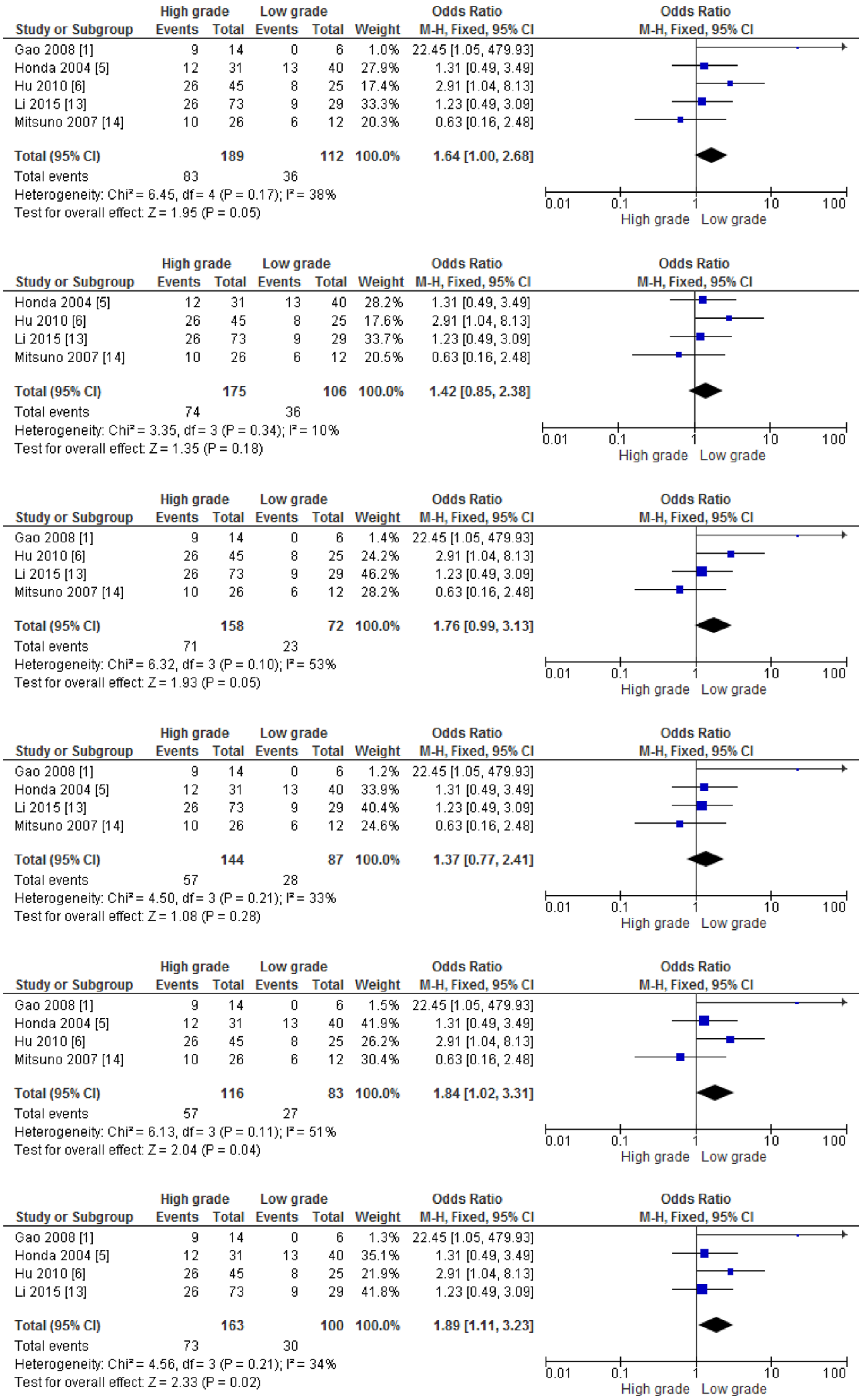

Supplementary Figure 2: Sensitivity analysis for *CHFR* promoter methylation in different grade of GC.

Study or Subgroup	Subserosa (+) Events	Total	Subserosa (-) Events	Total	Weight	Odds Ratio M-H, Fixed, 95% CI
Gao 2008 [1]	7	15	2	5	6.4%	1.31 [0.17, 10.26]
Hiraki 2010 [2]	7	11	11	20	11.4%	1.43 [0.32, 6.49]
Homma 2005 [4]	7	20	11	32	22.2%	1.03 [0.32, 3.32]
Li 2015 [13]	28	89	7	13	33.7%	0.39 [0.12, 1.28]
Oki 2009 [15]	12	36	8	23	26.2%	0.94 [0.31, 2.82]
**Total (95% CI)**		**171**		**93**	**100.0%**	**0.85 [0.48, 1.52]**
Total events	61		39			

Heterogeneity: Chi² = 2.40, df = 4 (P = 0.66); I² = 0%
Test for overall effect: Z = 0.53 (P = 0.59)

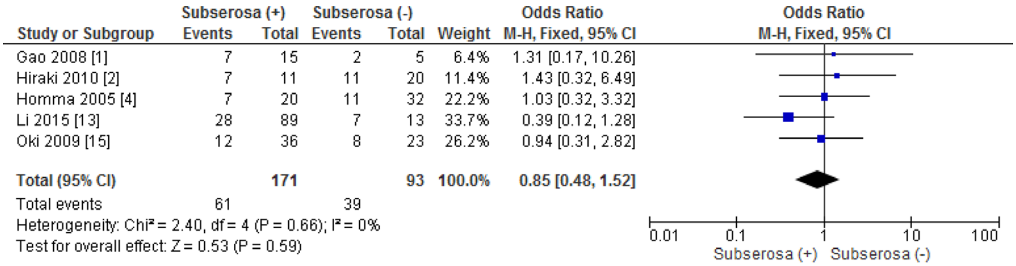

Study or Subgroup	Subserosa (+) Events	Total	Subserosa (-) Events	Total	Weight	Odds Ratio M-H, Fixed, 95% CI
Hiraki 2010 [2]	7	11	11	20	12.2%	1.43 [0.32, 6.49]
Homma 2005 [4]	7	20	11	32	23.7%	1.03 [0.32, 3.32]
Li 2015 [13]	28	89	7	13	36.1%	0.39 [0.12, 1.28]
Oki 2009 [15]	12	36	8	23	28.0%	0.94 [0.31, 2.82]
**Total (95% CI)**		**156**		**88**	**100.0%**	**0.82 [0.45, 1.50]**
Total events	54		37			

Heterogeneity: Chi² = 2.21, df = 3 (P = 0.53); I² = 0%
Test for overall effect: Z = 0.63 (P = 0.53)

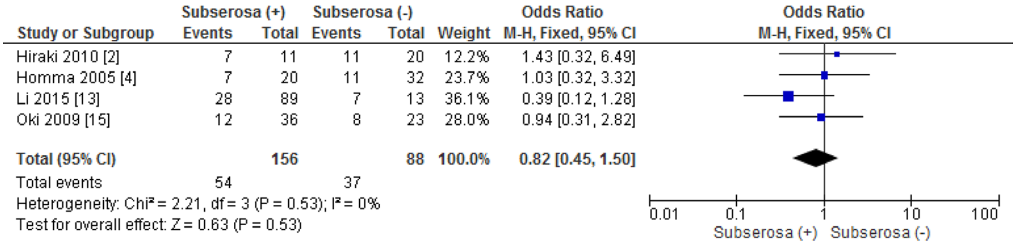

Study or Subgroup	Subserosa (+) Events	Total	Subserosa (-) Events	Total	Weight	Odds Ratio M-H, Fixed, 95% CI
Gao 2008 [1]	7	15	2	5	7.3%	1.31 [0.17, 10.26]
Homma 2005 [4]	7	20	11	32	25.0%	1.03 [0.32, 3.32]
Li 2015 [13]	28	89	7	13	38.1%	0.39 [0.12, 1.28]
Oki 2009 [15]	12	36	8	23	29.6%	0.94 [0.31, 2.82]
**Total (95% CI)**		**160**		**73**	**100.0%**	**0.78 [0.42, 1.46]**
Total events	54		28			

Heterogeneity: Chi² = 1.86, df = 3 (P = 0.60); I² = 0%
Test for overall effect: Z = 0.78 (P = 0.44)

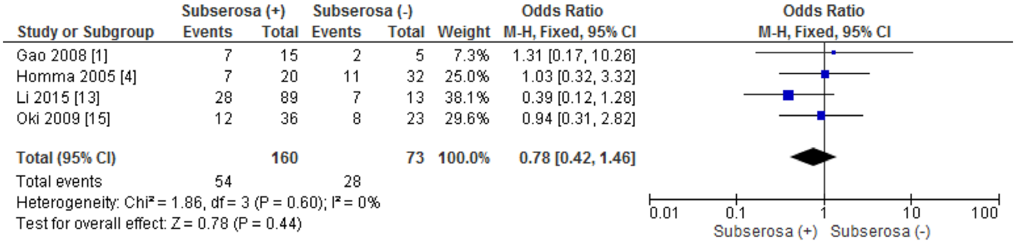

Study or Subgroup	Subserosa (+) Events	Total	Subserosa (-) Events	Total	Weight	Odds Ratio M-H, Fixed, 95% CI
Gao 2008 [1]	7	15	2	5	8.3%	1.31 [0.17, 10.26]
Hiraki 2010 [2]	7	11	11	20	14.7%	1.43 [0.32, 6.49]
Li 2015 [13]	28	89	7	13	43.3%	0.39 [0.12, 1.28]
Oki 2009 [15]	12	36	8	23	33.7%	0.94 [0.31, 2.82]
**Total (95% CI)**		**151**		**61**	**100.0%**	**0.81 [0.42, 1.56]**
Total events	54		28			

Heterogeneity: Chi² = 2.27, df = 3 (P = 0.52); I² = 0%
Test for overall effect: Z = 0.64 (P = 0.52)

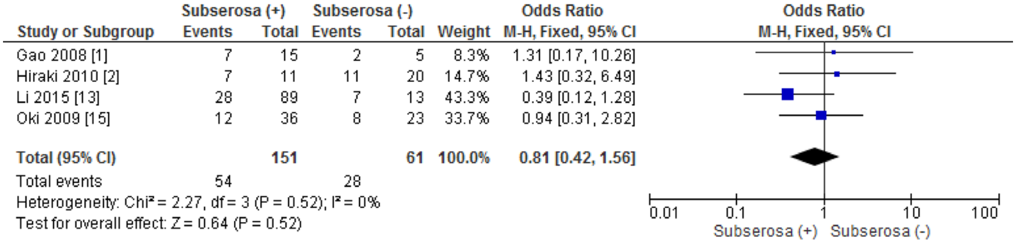

Study or Subgroup	Subserosa (+) Events	Total	Subserosa (-) Events	Total	Weight	Odds Ratio M-H, Fixed, 95% CI
Gao 2008 [1]	7	15	2	5	9.7%	1.31 [0.17, 10.26]
Hiraki 2010 [2]	7	11	11	20	17.3%	1.43 [0.32, 6.49]
Homma 2005 [4]	7	20	11	32	33.4%	1.03 [0.32, 3.32]
Oki 2009 [15]	12	36	8	23	39.6%	0.94 [0.31, 2.82]
**Total (95% CI)**		**82**		**80**	**100.0%**	**1.09 [0.56, 2.13]**
Total events	33		32			

Heterogeneity: Chi² = 0.24, df = 3 (P = 0.97); I² = 0%
Test for overall effect: Z = 0.25 (P = 0.80)

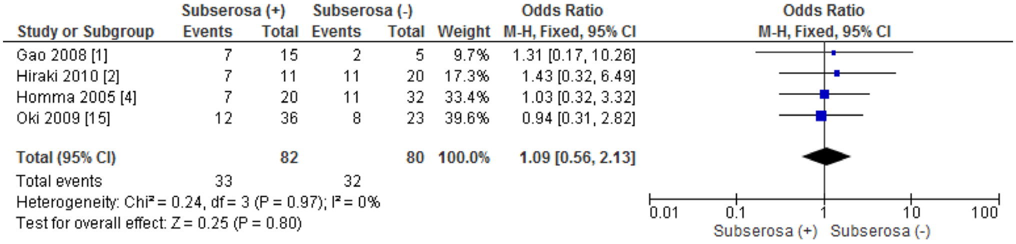

Study or Subgroup	Subserosa (+) Events	Total	Subserosa (-) Events	Total	Weight	Odds Ratio M-H, Fixed, 95% CI
Gao 2008 [1]	7	15	2	5	8.7%	1.31 [0.17, 10.26]
Hiraki 2010 [2]	7	11	11	20	15.5%	1.43 [0.32, 6.49]
Homma 2005 [4]	7	20	11	32	30.0%	1.03 [0.32, 3.32]
Li 2015 [13]	28	89	7	13	45.7%	0.39 [0.12, 1.28]
**Total (95% CI)**		**135**		**70**	**100.0%**	**0.83 [0.42, 1.63]**
Total events	49		31			

Heterogeneity: Chi² = 2.36, df = 3 (P = 0.50); I² = 0%
Test for overall effect: Z = 0.55 (P = 0.58)

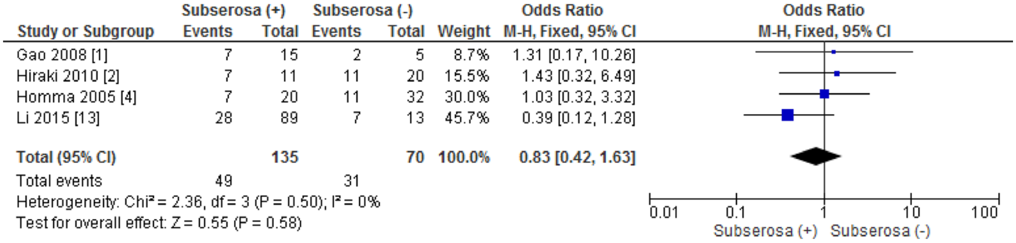

Supplementary Figure 3: Sensitivity analysis for *CHFR* promoter methylation in different invasion status of GC.

Study or Subgroup	Stage III/IV Events	Stage III/IV Total	Stage I/II Events	Stage I/II Total	Weight	Odds Ratio M-H, Fixed, 95% CI
Gao 2008 [1]	5	10	4	10	5.1%	1.50 [0.26, 8.82]
Hiraki 2010 [2]	15	25	16	24	16.7%	0.75 [0.23, 2.41]
Homma 2005 [4]	17	42	1	10	2.5%	6.12 [0.71, 52.85]
Honda 2004 [5]	19	56	6	15	16.0%	0.77 [0.24, 2.49]
Hu 2010 [6]	23	39	11	31	12.9%	2.61 [0.99, 6.92]
Li 2015 [13]	29	83	7	13	20.1%	0.46 [0.14, 1.50]
Mitsuno 2007 [14]	16	34	0	4	1.2%	8.03 [0.40, 160.61]
Morioka 2006 [9]	4	16	12	37	13.9%	0.69 [0.18, 2.61]
Oki 2009 [15]	15	42	5	17	11.7%	1.33 [0.39, 4.51]
**Total (95% CI)**		**347**		**161**	**100.0%**	**1.25 [0.82, 1.91]**
Total events	143		62			

Heterogeneity: Chi² = 10.73, df = 8 (P = 0.22); I² = 25%
Test for overall effect: Z = 1.04 (P = 0.30)

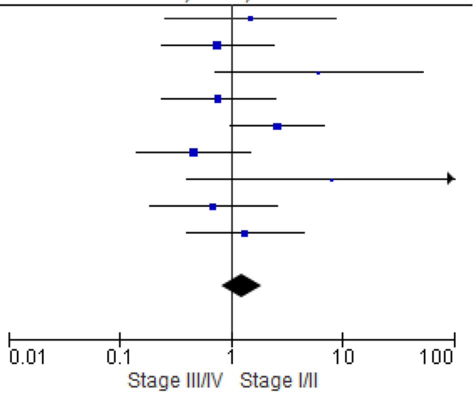

Study or Subgroup	Stage III/IV Events	Stage III/IV Total	Stage I/II Events	Stage I/II Total	Weight	Odds Ratio M-H, Fixed, 95% CI
Hiraki 2010 [2]	15	25	16	24	17.6%	0.75 [0.23, 2.41]
Homma 2005 [4]	17	42	1	10	2.6%	6.12 [0.71, 52.85]
Honda 2004 [5]	19	56	6	15	16.8%	0.77 [0.24, 2.49]
Hu 2010 [6]	23	39	11	31	13.5%	2.61 [0.99, 6.92]
Li 2015 [13]	29	83	7	13	21.2%	0.46 [0.14, 1.50]
Mitsuno 2007 [14]	16	34	0	4	1.2%	8.03 [0.40, 160.61]
Morioka 2006 [9]	4	16	12	37	14.6%	0.69 [0.18, 2.61]
Oki 2009 [15]	15	42	5	17	12.3%	1.33 [0.39, 4.51]
**Total (95% CI)**		**337**		**151**	**100.0%**	**1.24 [0.80, 1.91]**
Total events	138		58			

Heterogeneity: Chi² = 10.65, df = 7 (P = 0.15); I² = 34%
Test for overall effect: Z = 0.97 (P = 0.33)

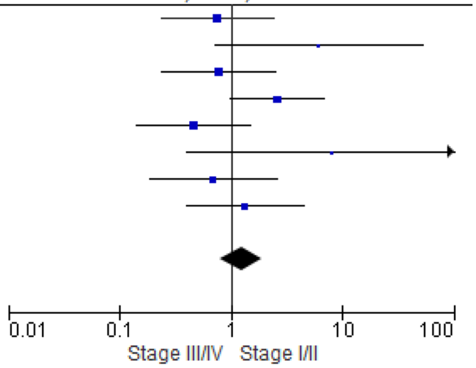

Study or Subgroup	Stage III/IV Events	Stage III/IV Total	Stage I/II Events	Stage I/II Total	Weight	Odds Ratio M-H, Fixed, 95% CI
Gao 2008 [1]	5	10	4	10	6.1%	1.50 [0.26, 8.82]
Homma 2005 [4]	17	42	1	10	3.0%	6.12 [0.71, 52.85]
Honda 2004 [5]	19	56	6	15	19.2%	0.77 [0.24, 2.49]
Hu 2010 [6]	23	39	11	31	15.4%	2.61 [0.99, 6.92]
Li 2015 [13]	29	83	7	13	24.2%	0.46 [0.14, 1.50]
Mitsuno 2007 [14]	16	34	0	4	1.4%	8.03 [0.40, 160.61]
Morioka 2006 [9]	4	16	12	37	16.7%	0.69 [0.18, 2.61]
Oki 2009 [15]	15	42	5	17	14.0%	1.33 [0.39, 4.51]
**Total (95% CI)**		**322**		**137**	**100.0%**	**1.35 [0.86, 2.13]**
Total events	128		46			

Heterogeneity: Chi² = 10.07, df = 7 (P = 0.18); I² = 31%
Test for overall effect: Z = 1.30 (P = 0.19)

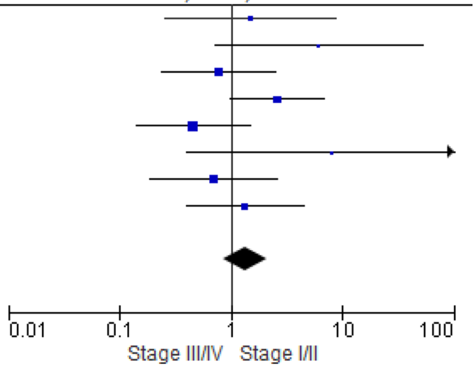

Study or Subgroup	Stage III/IV Events	Stage III/IV Total	Stage I/II Events	Stage I/II Total	Weight	Odds Ratio M-H, Fixed, 95% CI
Gao 2008 [1]	5	10	4	10	5.2%	1.50 [0.26, 8.82]
Hiraki 2010 [2]	15	25	16	24	17.1%	0.75 [0.23, 2.41]
Honda 2004 [5]	19	56	6	15	16.4%	0.77 [0.24, 2.49]
Hu 2010 [6]	23	39	11	31	13.2%	2.61 [0.99, 6.92]
Li 2015 [13]	29	83	7	13	20.6%	0.46 [0.14, 1.50]
Mitsuno 2007 [14]	16	34	0	4	1.2%	8.03 [0.40, 160.61]
Morioka 2006 [9]	4	16	12	37	14.2%	0.69 [0.18, 2.61]
Oki 2009 [15]	15	42	5	17	12.0%	1.33 [0.39, 4.51]
**Total (95% CI)**		**305**		**151**	**100.0%**	**1.13 [0.73, 1.75]**
Total events	126		61			

Heterogeneity: Chi² = 8.29, df = 7 (P = 0.31); I² = 16%
Test for overall effect: Z = 0.54 (P = 0.59)

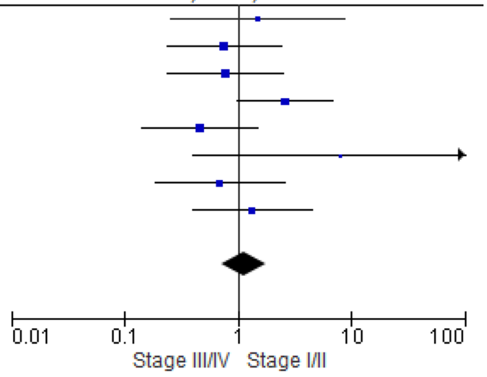

Study or Subgroup	Stage III/IV Events	Stage III/IV Total	Stage I/II Events	Stage I/II Total	Weight	Odds Ratio M-H, Fixed, 95% CI
Gao 2008 [1]	5	10	4	10	6.1%	1.50 [0.26, 8.82]
Hiraki 2010 [2]	15	25	16	24	19.9%	0.75 [0.23, 2.41]
Homma 2005 [4]	17	42	1	10	2.9%	6.12 [0.71, 52.85]
Hu 2010 [6]	23	39	11	31	15.3%	2.61 [0.99, 6.92]
Li 2015 [13]	29	83	7	13	24.0%	0.46 [0.14, 1.50]
Mitsuno 2007 [14]	16	34	0	4	1.4%	8.03 [0.40, 160.61]
Morioka 2006 [9]	4	16	12	37	16.5%	0.69 [0.18, 2.61]
Oki 2009 [15]	15	42	5	17	13.9%	1.33 [0.39, 4.51]
**Total (95% CI)**		**291**		**146**	**100.0%**	**1.34 [0.86, 2.11]**
Total events	124		56			

Heterogeneity: Chi² = 10.15, df = 7 (P = 0.18); I² = 31%
Test for overall effect: Z = 1.28 (P = 0.20)

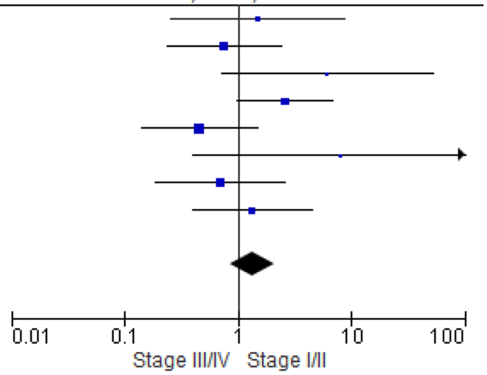

Study or Subgroup	Stage III/IV Events	Stage III/IV Total	Stage I/II Events	Stage I/II Total	Weight	Odds Ratio M-H, Fixed, 95% CI
Gao 2008 [1]	5	10	4	10	5.9%	1.50 [0.26, 8.82]
Hiraki 2010 [2]	15	25	16	24	19.2%	0.75 [0.23, 2.41]
Homma 2005 [4]	17	42	1	10	2.8%	6.12 [0.71, 52.85]
Honda 2004 [5]	19	56	6	15	18.3%	0.77 [0.24, 2.49]
Li 2015 [13]	29	83	7	13	23.1%	0.46 [0.14, 1.50]
Mitsuno 2007 [14]	16	34	0	4	1.4%	8.03 [0.40, 160.61]
Morioka 2006 [9]	4	16	12	37	15.9%	0.69 [0.18, 2.61]
Oki 2009 [15]	15	42	5	17	13.4%	1.33 [0.39, 4.51]
**Total (95% CI)**		**308**		**130**	**100.0%**	**1.05 [0.66, 1.68]**
Total events	120		51			

Heterogeneity: Chi² = 7.48, df = 7 (P = 0.38); I² = 6%
Test for overall effect: Z = 0.21 (P = 0.84)

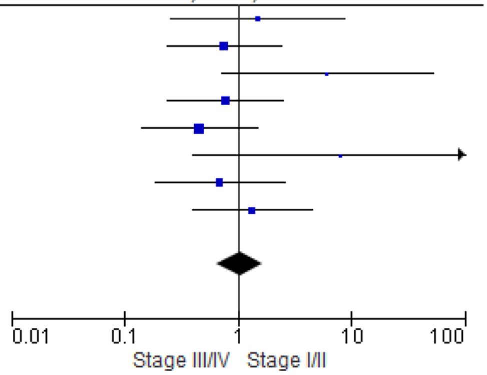

Study or Subgroup	Stage III/IV Events	Stage III/IV Total	Stage I/II Events	Stage I/II Total	Weight	Odds Ratio M-H, Fixed, 95% CI
Gao 2008 [1]	5	10	4	10	6.4%	1.50 [0.26, 8.82]
Hiraki 2010 [2]	15	25	16	24	20.9%	0.75 [0.23, 2.41]
Homma 2005 [4]	17	42	1	10	3.1%	6.12 [0.71, 52.85]
Honda 2004 [5]	19	56	6	15	20.0%	0.77 [0.24, 2.49]
Hu 2010 [6]	23	39	11	31	16.1%	2.61 [0.99, 6.92]
Mitsuno 2007 [14]	16	34	0	4	1.5%	8.03 [0.40, 160.61]
Morioka 2006 [9]	4	16	12	37	17.4%	0.69 [0.18, 2.61]
Oki 2009 [15]	15	42	5	17	14.6%	1.33 [0.39, 4.51]
**Total (95% CI)**		**264**		**148**	**100.0%**	**1.45 [0.92, 2.29]**
Total events	114		55			

Heterogeneity: Chi² = 7.93, df = 7 (P = 0.34); I² = 12%
Test for overall effect: Z = 1.60 (P = 0.11)

Odds Ratio M-H, Fixed, 95% CI

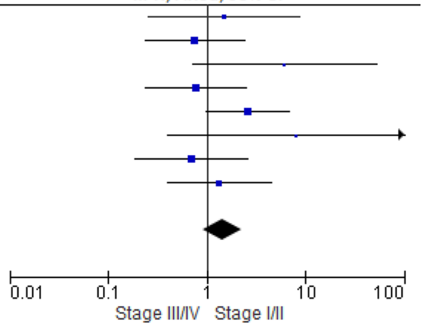

Study or Subgroup	Stage III/IV Events	Stage III/IV Total	Stage I/II Events	Stage I/II Total	Weight	Odds Ratio M-H, Fixed, 95% CI
Gao 2008 [1]	5	10	4	10	5.2%	1.50 [0.26, 8.82]
Hiraki 2010 [2]	15	25	16	24	16.9%	0.75 [0.23, 2.41]
Homma 2005 [4]	17	42	1	10	2.5%	6.12 [0.71, 52.85]
Honda 2004 [5]	19	56	6	15	16.2%	0.77 [0.24, 2.49]
Hu 2010 [6]	23	39	11	31	13.0%	2.61 [0.99, 6.92]
Li 2015 [13]	29	83	7	13	20.4%	0.46 [0.14, 1.50]
Morioka 2006 [9]	4	16	12	37	14.1%	0.69 [0.18, 2.61]
Oki 2009 [15]	15	42	5	17	11.8%	1.33 [0.39, 4.51]
**Total (95% CI)**		**313**		**157**	**100.0%**	**1.17 [0.76, 1.80]**
Total events	127		62			

Heterogeneity: Chi² = 9.04, df = 7 (P = 0.25); I² = 23%
Test for overall effect: Z = 0.72 (P = 0.47)

Odds Ratio M-H, Fixed, 95% CI

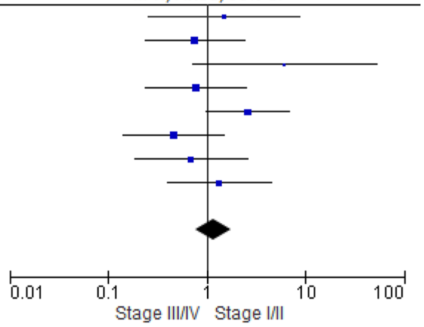

Study or Subgroup	Stage III/IV Events	Stage III/IV Total	Stage I/II Events	Stage I/II Total	Weight	Odds Ratio M-H, Fixed, 95% CI
Gao 2008 [1]	5	10	4	10	5.9%	1.50 [0.26, 8.82]
Hiraki 2010 [2]	15	25	16	24	19.4%	0.75 [0.23, 2.41]
Homma 2005 [4]	17	42	1	10	2.9%	6.12 [0.71, 52.85]
Honda 2004 [5]	19	56	6	15	18.6%	0.77 [0.24, 2.49]
Hu 2010 [6]	23	39	11	31	14.9%	2.61 [0.99, 6.92]
Li 2015 [13]	29	83	7	13	23.4%	0.46 [0.14, 1.50]
Mitsuno 2007 [14]	16	34	0	4	1.4%	8.03 [0.40, 160.61]
Oki 2009 [15]	15	42	5	17	13.6%	1.33 [0.39, 4.51]
**Total (95% CI)**		**331**		**124**	**100.0%**	**1.34 [0.86, 2.10]**
Total events	139		50			

Heterogeneity: Chi² = 10.06, df = 7 (P = 0.19); I² = 30%
Test for overall effect: Z = 1.29 (P = 0.20)

Odds Ratio M-H, Fixed, 95% CI

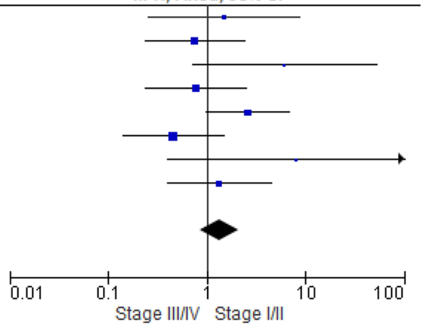

Study or Subgroup	Stage III/IV Events	Stage III/IV Total	Stage I/II Events	Stage I/II Total	Weight	Odds Ratio M-H, Fixed, 95% CI
Gao 2008 [1]	5	10	4	10	5.8%	1.50 [0.26, 8.82]
Hiraki 2010 [2]	15	25	16	24	18.9%	0.75 [0.23, 2.41]
Homma 2005 [4]	17	42	1	10	2.8%	6.12 [0.71, 52.85]
Honda 2004 [5]	19	56	6	15	18.1%	0.77 [0.24, 2.49]
Hu 2010 [6]	23	39	11	31	14.6%	2.61 [0.99, 6.92]
Li 2015 [13]	29	83	7	13	22.8%	0.46 [0.14, 1.50]
Mitsuno 2007 [14]	16	34	0	4	1.3%	8.03 [0.40, 160.61]
Morioka 2006 [9]	4	16	12	37	15.7%	0.69 [0.18, 2.61]
**Total (95% CI)**		**305**		**144**	**100.0%**	**1.24 [0.79, 1.94]**
Total events	128		57			

Heterogeneity: Chi² = 10.69, df = 7 (P = 0.15); I² = 35%
Test for overall effect: Z = 0.94 (P = 0.35)

Odds Ratio M-H, Fixed, 95% CI

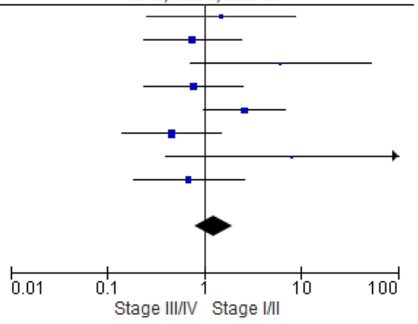

Supplementary Figure 4: Sensitivity analysis for *CHFR* promoter methylation in GC stage III/IV and stage I/II.

Study or Subgroup	LN (+) Events	LN (+) Total	LN (-) Events	LN (-) Total	Weight	Odds Ratio M-H, Fixed, 95% CI
Gao 2008 [1]	25	66	10	36	20.0%	1.59 [0.66, 3.83]
Hiraki 2010 [2]	20	34	11	15	15.7%	0.52 [0.14, 1.97]
Honda 2004 [5]	20	58	5	13	13.3%	0.84 [0.24, 2.91]
Hu 2010 [6]	27	47	7	23	10.0%	3.09 [1.07, 8.90]
Li 2015 [13]	27	47	7	23	10.0%	3.09 [1.07, 8.90]
Mitsuno 2007 [14]	12	28	4	10	8.4%	1.13 [0.26, 4.89]
Morioka 2006 [9]	9	28	7	25	12.5%	1.22 [0.37, 3.96]
Oki 2009 [15]	15	39	5	20	10.1%	1.88 [0.56, 6.23]
**Total (95% CI)**		**347**		**165**	**100.0%**	**1.56 [1.05, 2.32]**
Total events	155		56			

Heterogeneity: Chi² = 7.20, df = 7 (P = 0.41); I² = 3%
Test for overall effect: Z = 2.22 (P = 0.03)

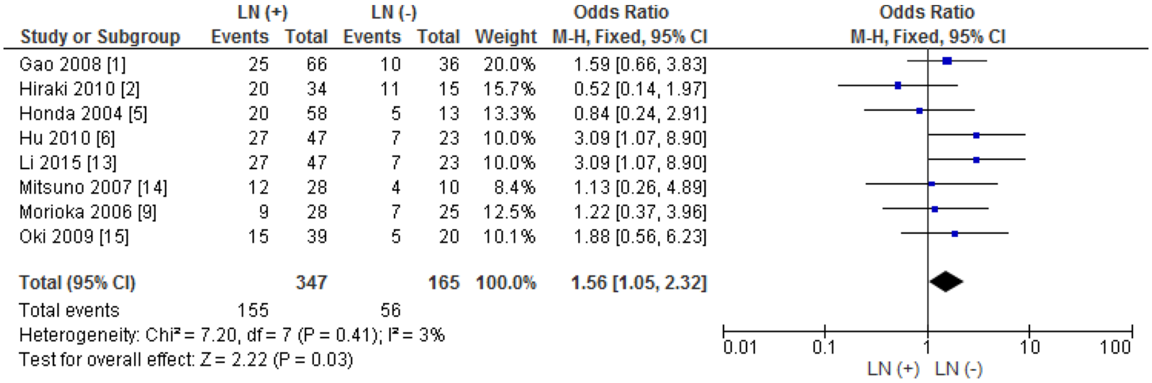

Study or Subgroup	LN (+) Events	LN (+) Total	LN (-) Events	LN (-) Total	Weight	Odds Ratio M-H, Fixed, 95% CI
Hiraki 2010 [2]	20	34	11	15	19.6%	0.52 [0.14, 1.97]
Honda 2004 [5]	20	58	5	13	16.7%	0.84 [0.24, 2.91]
Hu 2010 [6]	27	47	7	23	12.5%	3.09 [1.07, 8.90]
Li 2015 [13]	27	47	7	23	12.5%	3.09 [1.07, 8.90]
Mitsuno 2007 [14]	12	28	4	10	10.5%	1.13 [0.26, 4.89]
Morioka 2006 [9]	9	28	7	25	15.6%	1.22 [0.37, 3.96]
Oki 2009 [15]	15	39	5	20	12.7%	1.88 [0.56, 6.23]
**Total (95% CI)**		**281**		**129**	**100.0%**	**1.56 [1.00, 2.42]**
Total events	130		46			

Heterogeneity: Chi² = 7.19, df = 6 (P = 0.30); I² = 17%
Test for overall effect: Z = 1.97 (P = 0.05)

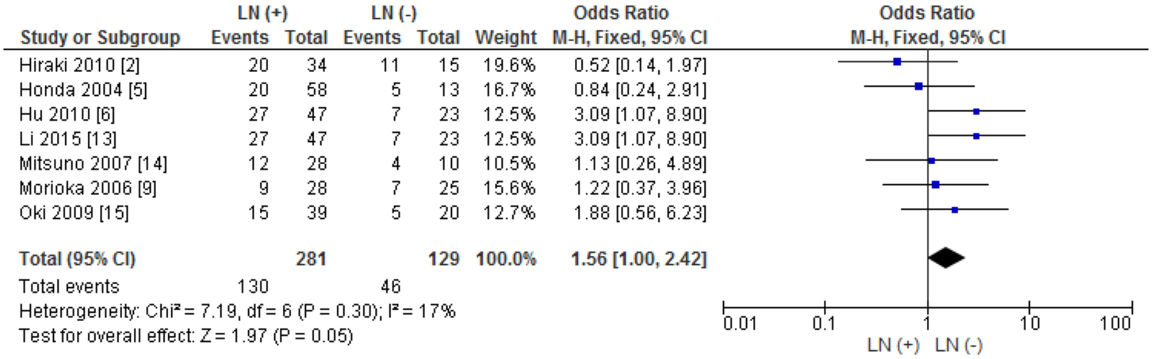

Study or Subgroup	LN (+) Events	LN (+) Total	LN (-) Events	LN (-) Total	Weight	Odds Ratio M-H, Fixed, 95% CI
Gao 2008 [1]	25	66	10	36	23.8%	1.59 [0.66, 3.83]
Honda 2004 [5]	20	58	5	13	15.8%	0.84 [0.24, 2.91]
Hu 2010 [6]	27	47	7	23	11.8%	3.09 [1.07, 8.90]
Li 2015 [13]	27	47	7	23	11.8%	3.09 [1.07, 8.90]
Mitsuno 2007 [14]	12	28	4	10	10.0%	1.13 [0.26, 4.89]
Morioka 2006 [9]	9	28	7	25	14.8%	1.22 [0.37, 3.96]
Oki 2009 [15]	15	39	5	20	12.0%	1.88 [0.56, 6.23]
**Total (95% CI)**		**313**		**150**	**100.0%**	**1.76 [1.16, 2.67]**
Total events	135		45			

Heterogeneity: Chi² = 4.30, df = 6 (P = 0.64); I² = 0%
Test for overall effect: Z = 2.64 (P = 0.008)

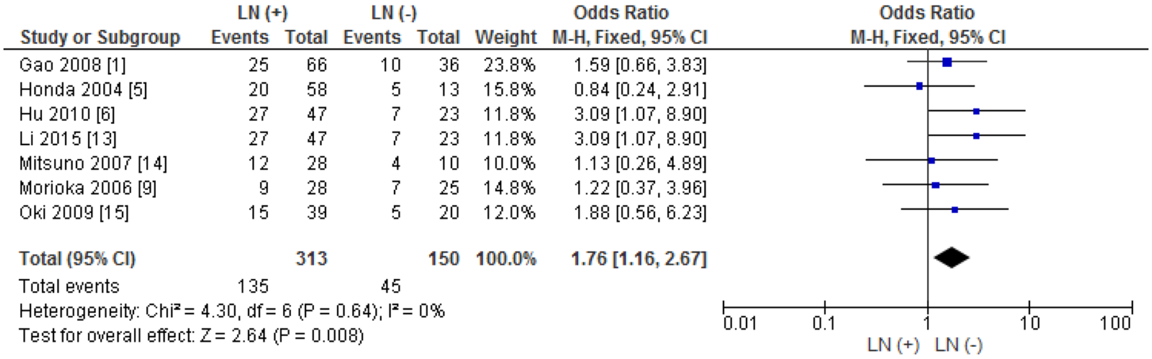

Study or Subgroup	LN (+) Events	LN (+) Total	LN (-) Events	LN (-) Total	Weight	Odds Ratio M-H, Fixed, 95% CI
Gao 2008 [1]	25	66	10	36	23.1%	1.59 [0.66, 3.83]
Hiraki 2010 [2]	20	34	11	15	18.1%	0.52 [0.14, 1.97]
Hu 2010 [6]	27	47	7	23	11.5%	3.09 [1.07, 8.90]
Li 2015 [13]	27	47	7	23	11.5%	3.09 [1.07, 8.90]
Mitsuno 2007 [14]	12	28	4	10	9.7%	1.13 [0.26, 4.89]
Morioka 2006 [9]	9	28	7	25	14.4%	1.22 [0.37, 3.96]
Oki 2009 [15]	15	39	5	20	11.7%	1.88 [0.56, 6.23]
**Total (95% CI)**		**289**		**152**	**100.0%**	**1.67 [1.10, 2.54]**
Total events	135		51			

Heterogeneity: Chi² = 6.13, df = 6 (P = 0.41); I² = 2%
Test for overall effect: Z = 2.43 (P = 0.02)

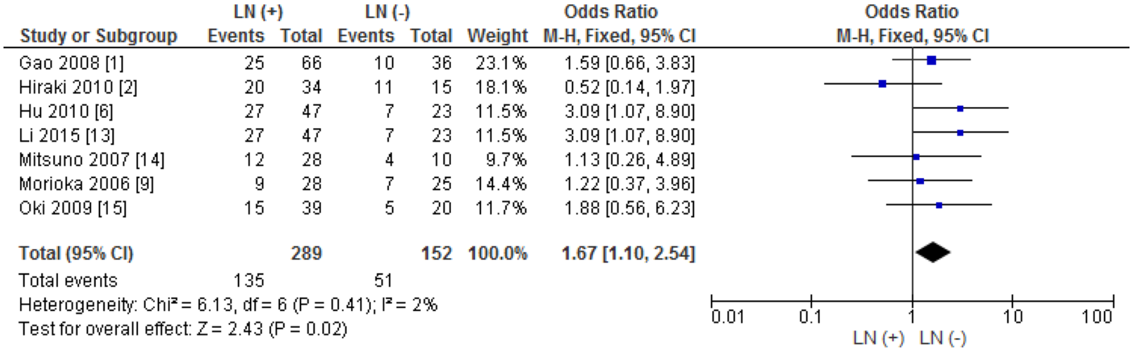

Study or Subgroup	LN (+) Events	LN (+) Total	LN (-) Events	LN (-) Total	Weight	Odds Ratio M-H, Fixed, 95% CI
Gao 2008 [1]	25	66	10	36	22.2%	1.59 [0.66, 3.83]
Hiraki 2010 [2]	20	34	11	15	17.4%	0.52 [0.14, 1.97]
Honda 2004 [5]	20	58	5	13	14.8%	0.84 [0.24, 2.91]
Li 2015 [13]	27	47	7	23	11.1%	3.09 [1.07, 8.90]
Mitsuno 2007 [14]	12	28	4	10	9.3%	1.13 [0.26, 4.89]
Morioka 2006 [9]	9	28	7	25	13.9%	1.22 [0.37, 3.96]
Oki 2009 [15]	15	39	5	20	11.3%	1.88 [0.56, 6.23]
**Total (95% CI)**		**300**		**142**	**100.0%**	**1.39 [0.91, 2.14]**
Total events	128		49			

Heterogeneity: Chi² = 5.35, df = 6 (P = 0.50); I² = 0%
Test for overall effect: Z = 1.53 (P = 0.13)

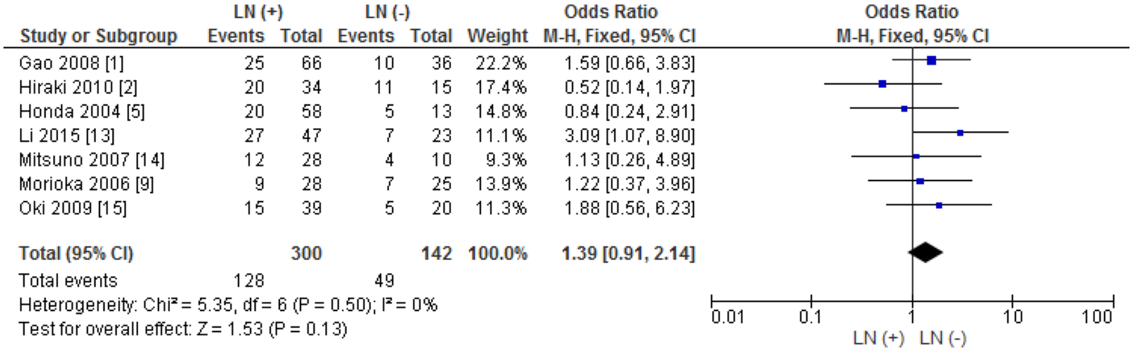

Study or Subgroup	LN (+) Events	LN (+) Total	LN (-) Events	LN (-) Total	Weight	Odds Ratio M-H, Fixed, 95% CI
Gao 2008 [1]	25	66	10	36	22.2%	1.59 [0.66, 3.83]
Hiraki 2010 [2]	20	34	11	15	17.4%	0.52 [0.14, 1.97]
Honda 2004 [5]	20	58	5	13	14.8%	0.84 [0.24, 2.91]
Hu 2010 [6]	27	47	7	23	11.1%	3.09 [1.07, 8.90]
Mitsuno 2007 [14]	12	28	4	10	9.3%	1.13 [0.26, 4.89]
Morioka 2006 [9]	9	28	7	25	13.9%	1.22 [0.37, 3.96]
Oki 2009 [15]	15	39	5	20	11.3%	1.88 [0.56, 6.23]
**Total (95% CI)**		**300**		**142**	**100.0%**	**1.39 [0.91, 2.14]**
Total events	128		49			

Heterogeneity: Chi² = 5.35, df = 6 (P = 0.50); I² = 0%
Test for overall effect: Z = 1.53 (P = 0.13)

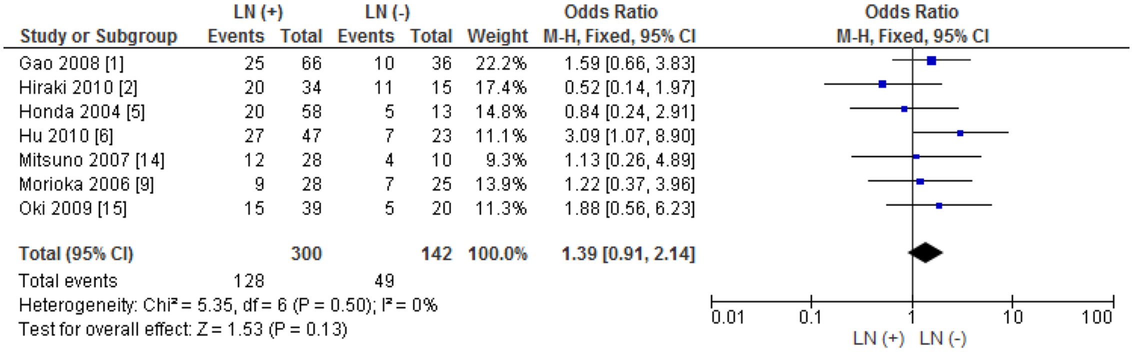

Study or Subgroup	LN (+) Events	LN (+) Total	LN (-) Events	LN (-) Total	Weight	Odds Ratio M-H, Fixed, 95% CI
Gao 2008 [1]	25	66	10	36	21.9%	1.59 [0.66, 3.83]
Hiraki 2010 [2]	20	34	11	15	17.1%	0.52 [0.14, 1.97]
Honda 2004 [5]	20	58	5	13	14.6%	0.84 [0.24, 2.91]
Hu 2010 [6]	27	47	7	23	10.9%	3.09 [1.07, 8.90]
Li 2015 [13]	27	47	7	23	10.9%	3.09 [1.07, 8.90]
Morioka 2006 [9]	9	28	7	25	13.7%	1.22 [0.37, 3.96]
Oki 2009 [15]	15	39	5	20	11.1%	1.88 [0.56, 6.23]
**Total (95% CI)**		**319**		**155**	**100.0%**	**1.60 [1.06, 2.41]**
Total events	143		52			

Heterogeneity: Chi² = 6.99, df = 6 (P = 0.32); I² = 14%
Test for overall effect: Z = 2.26 (P = 0.02)

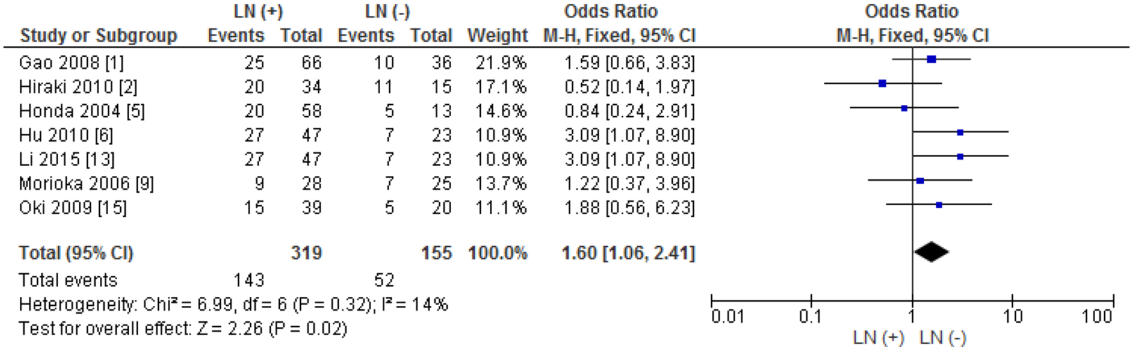

Study or Subgroup	LN (+) Events	LN (+) Total	LN (-) Events	LN (-) Total	Weight	Odds Ratio M-H, Fixed, 95% CI
Gao 2008 [1]	25	66	10	36	22.9%	1.59 [0.66, 3.83]
Hiraki 2010 [2]	20	34	11	15	17.9%	0.52 [0.14, 1.97]
Honda 2004 [5]	20	58	5	13	15.2%	0.84 [0.24, 2.91]
Hu 2010 [6]	27	47	7	23	11.4%	3.09 [1.07, 8.90]
Li 2015 [13]	27	47	7	23	11.4%	3.09 [1.07, 8.90]
Mitsuno 2007 [14]	12	28	4	10	9.6%	1.13 [0.26, 4.89]
Oki 2009 [15]	15	39	5	20	11.6%	1.88 [0.56, 6.23]
**Total (95% CI)**		**319**		**140**	**100.0%**	**1.61 [1.06, 2.45]**
Total events	146		49			

Heterogeneity: Chi² = 7.00, df = 6 (P = 0.32); I² = 14%
Test for overall effect: Z = 2.24 (P = 0.03)

Odds Ratio
M-H, Fixed, 95% CI

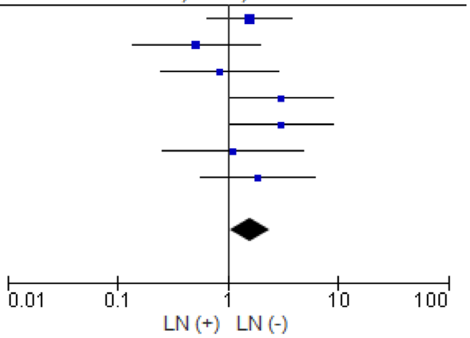

Study or Subgroup	LN (+) Events	LN (+) Total	LN (-) Events	LN (-) Total	Weight	Odds Ratio M-H, Fixed, 95% CI
Gao 2008 [1]	25	66	10	36	22.3%	1.59 [0.66, 3.83]
Hiraki 2010 [2]	20	34	11	15	17.4%	0.52 [0.14, 1.97]
Honda 2004 [5]	20	58	5	13	14.8%	0.84 [0.24, 2.91]
Hu 2010 [6]	27	47	7	23	11.1%	3.09 [1.07, 8.90]
Li 2015 [13]	27	47	7	23	11.1%	3.09 [1.07, 8.90]
Mitsuno 2007 [14]	12	28	4	10	9.3%	1.13 [0.26, 4.89]
Morioka 2006 [9]	9	28	7	25	13.9%	1.22 [0.37, 3.96]
**Total (95% CI)**		**308**		**145**	**100.0%**	**1.53 [1.01, 2.32]**
Total events	140		51			

Heterogeneity: Chi² = 7.10, df = 6 (P = 0.31); I² = 15%
Test for overall effect: Z = 1.99 (P = 0.05)

Odds Ratio
M-H, Fixed, 95% CI

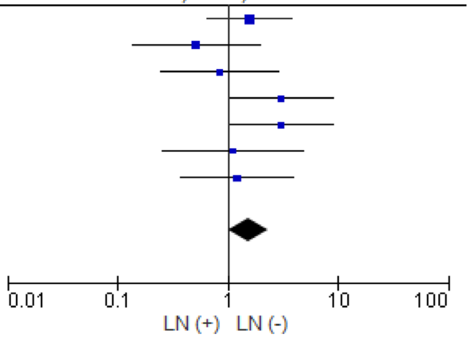

Supplementary Figure 5: Sensitivity analysis for *CHFR* promoter methylation in different status of lymph node metastasis.

REFERENCES

1. Gao YJ, Xin Y, Zhang JJ, Zhou J. Mechanism and pathobiologic implications of CHFR promoter methylation in gastric carcinoma. World J Gastroenterol. 2008; 14:5000–07. https://doi.org/10.3748/wjg.14.5000.
2. Hiraki M, Kitajima Y, Sato S, Mitsuno M, Koga Y, Nakamura J, Hashiguchi K, Noshiro H, Miyazaki K. Aberrant gene methylation in the lymph nodes provides a possible marker for diagnosing micrometastasis in gastric cancer. Ann Surg Oncol. 2010; 17:1177–86. https://doi.org/10.1245/s10434-009-0815-8.
3. Hiraki M, Kitajima Y, Koga Y, Tanaka T, Nakamura J, Hashiguchi K, Noshiro H, Miyazaki K. Aberrant gene methylation is a biomarker for the detection of cancer cells in peritoneal wash samples from advanced gastric cancer patients. Ann Surg Oncol. 2011; 18:3013–19. https://doi.org/10.1245/s10434-011-1636-0.
4. Homma N, Tamura G, Honda T, Jin Z, Ohmura K, Kawata S, Motoyama T. Hypermethylation of Chfr and hMLH1 in gastric noninvasive and early invasive neoplasias. Virchows Arch. 2005; 446:120–26. https://doi.org/10.1007/s00428-004-1146-6.
5. Honda T, Tamura G, Waki T, Kawata S, Nishizuka S, Motoyama T. Promoter hypermethylation of the Chfr gene in neoplastic and non-neoplastic gastric epithelia. Br J Cancer. 2004; 90:2013–16. https://doi.org/10.1038/sj.bjc.6601849.
6. Hu SL, Kong XY, Cheng ZD, Sun YB, Shen G, Xu WP, Wu L, Xu XC, Jiang XD, Huang DB. Promoter methylation of p16, Runx3, DAPK and CHFR genes is frequent in gastric carcinoma. Tumori. 2010; 96:726–33.
7. Kang GH, Lee S, Cho NY, Gandamihardja T, Long TI, Weisenberger DJ, Campan M, Laird PW. DNA methylation profiles of gastric carcinoma characterized by quantitative DNA methylation analysis. Lab Invest. 2008; 88:161–70. https://doi.org/10.1038/labinvest.3700707.
8. Koga Y, Kitajima Y, Miyoshi A, Sato K, Sato S, Miyazaki K. The significance of aberrant CHFR methylation for clinical response to microtubule inhibitors in gastric cancer. J Gastroenterol. 2006; 41:133–39. https://doi.org/10.1007/s00535-005-1732-7.
9. Morioka Y, Hibi K, Sakai M, Koike M, Fujiwara M, Kodera Y, Ito K, Nakao A. Aberrant methylation of the CHFR gene in digestive tract cancer. Anticancer Res. 2006; 26:1791–95.
10. Satoh A, Toyota M, Itoh F, Sasaki Y, Suzuki H, Ogi K, Kikuchi T, Mita H, Yamashita T, Kojima T, Kusano M, Fujita M, Hosokawa M, et al. Epigenetic inactivation of CHFR and sensitivity to microtubule inhibitors in gastric cancer. Cancer Res. 2003; 63:8606–13.
11. Wang M, Shen L, Deng D. Association between CHFR methylation and chemosensitivity of paclitaxel in advanced gastric cancer. Med Oncol. 2014; 31:907. https://doi.org/10.1007/s12032-014-0907-6.
12. Yoshida K, Hamai Y, Suzuki T, Sanada Y, Oue N, Yasui W. DNA methylation of CHFR is not a predictor of the response to docetaxel and paclitaxel in advanced and recurrent gastric cancer. Anticancer Res. 2006; 26:49–54.
13. Li Y, Yang Y, Lu Y, Herman JG, Brock MV, Zhao P, Guo M. Predictive value of CHFR and MLH1 methylation in human gastric cancer. Gastric Cancer. 2015; 18:280–87. https://doi.org/10.1007/s10120-014-0370-2.
14. Mitsuno M, Kitajima Y, Ide T, Ohtaka K, Tanaka M, Satoh S, Miyazaki K. Aberrant methylation of p16 predicts candidates for 5-fluorouracil-based adjuvant therapy in gastric cancer patients. J Gastroenterol. 2007; 42:866–73. https://doi.org/10.1007/s00535-007-2113-1.
15. Oki E, Zhao Y, Yoshida R, Masuda T, Ando K, Sugiyama M, Tokunaga E, Morita M, Kakeji Y, Maehara Y. Checkpoint with forkhead-associated and ring finger promoter hypermethylation correlates with microsatellite instability in gastric cancer. World J Gastroenterol. 2009; 15:2520–25. https://doi.org/10.3748/wjg.15.2520.
